# Scallop genome reveals molecular adaptations to semi-sessile life and neurotoxins

**DOI:** 10.1038/s41467-017-01927-0

**Published:** 2017-11-23

**Authors:** Yuli Li, Xiaoqing Sun, Xiaoli Hu, Xiaogang Xun, Jinbo Zhang, Ximing Guo, Wenqian Jiao, Lingling Zhang, Weizhi Liu, Jing Wang, Ji Li, Yan Sun, Yan Miao, Xiaokang Zhang, Taoran Cheng, Guoliang Xu, Xiaoteng Fu, Yangfan Wang, Xinran Yu, Xiaoting Huang, Wei Lu, Jia Lv, Chuang Mu, Dawei Wang, Xu Li, Yu Xia, Yajuan Li, Zhihui Yang, Fengliang Wang, Lu Zhang, Qiang Xing, Huaiqian Dou, Xianhui Ning, Jinzhuang Dou, Yangping Li, Dexu Kong, Yaran Liu, Zhi Jiang, Ruiqiang Li, Shi Wang, Zhenmin Bao

**Affiliations:** 1 Key Laboratory of Marine Genetics and Breeding (Ministry of Education), Ocean University of China, Qingdao, 266003 China; 2 Novogene Bioinformatics Institute, Beijing, 100083 China; 3 Laboratory for Marine Fisheries Science and Food Production Processes, Qingdao National Laboratory for Marine Science and Technology, Qingdao, 266237 China; 4 Haskin Shellfish Research Laboratory, Department of Marine and Coastal Sciences, Rutgers University, Port Norris, NJ 08349 USA; 5 Laboratory for Marine Biology and Biotechnology, Qingdao National Laboratory for Marine Science and Technology, Qingdao, 266237 China

## Abstract

Bivalve molluscs are descendants of an early-Cambrian lineage superbly adapted to benthic filter feeding. Adaptations in form and behavior are well recognized, but the underlying molecular mechanisms are largely unknown. Here, we investigate the genome, various transcriptomes, and proteomes of the scallop *Chlamys farreri*, a semi-sessile bivalve with well-developed adductor muscle, sophisticated eyes, and remarkable neurotoxin resistance. The scallop’s large striated muscle is energy-dynamic but not fully differentiated from smooth muscle. Its eyes are supported by highly diverse, intronless opsins expanded by retroposition for broadened spectral sensitivity. Rapid byssal secretion is enabled by a specialized foot and multiple proteins including expanded tyrosinases. The scallop uses hepatopancreas to accumulate neurotoxins and kidney to transform to high-toxicity forms through expanded sulfotransferases, probably as deterrence against predation, while it achieves neurotoxin resistance through point mutations in sodium channels. These findings suggest that expansion and mutation of those genes may have profound effects on scallop’s phenotype and adaptation.

## Abstract (web summary)

Bivalve molluscs have evolved various characteristics to adapt to benthic filter-feeding. Here, Li et al investigate the genome, transcriptomes and proteomes of scallop *Chlamys farreri*, revealing evidences of molecular adaptations to semi-sessile life and neurotoxins.

## Introduction

Bivalve molluscs, which first appeared in the early-Cambrian over 500 million years ago (MYA), represent an ancient lineage of bilaterians that has survived several mass extinction events^1^. Yet, extant bivalves with ~9600 species^2^ remain abundant and thriving in world oceans and freshwater environments ranging from tropical to polar regions and from intertidal zones to deep seas. Bivalves are well adapted to benthic life as sessile, semi-sessile, or free-living filter feeders and play critical roles in benthic ecology. Many bivalves are important fishery and aquaculture species providing significant economic benefits to humans. Despite their biological, ecological, and economic significance, their genomes are poorly sampled for whole-genome studies^3–7^, limiting our understanding of bilaterian evolution, especially molecular adaptations in this ancient but successful lineage.

Scallops are among the best-known bivalves recognized for their beautiful shells of elegant shapes, sophisticated patterns, and diverse colors. Scallops have some unique characteristics making them good models to study development, adaptation, and early animal evolution as indicated by a recent genome analysis of Yesso scallop *Patinopecten yessoensis* ^5^. Scallops have a large adductor muscle, probably as an adaptation to swimming by clapping valves for avoiding predation and seeking favorable habitats^8^. They are rare among lophotrochozoans in having numerous image-forming eyes along the edges of their mantles that perform vital functions in detecting predators and guiding swimming^9^. Scallops can attach to substrates as juveniles by rapidly producing adhesive and strong byssal threads that are either retained or lost in adults. As filter feeders that may feed on toxic dinoflagellates, scallops can accumulate and tolerate high levels of neurotoxins such as paralytic shellfish toxins (PSTs) that are among the most potent natural toxins for humans^10, 11^. These adaptive features are remarkable, and understanding the molecular innovations underlying these remarkable features may provide insights into how organisms adapt to their environments and evolve, which is a fundamental question in evolutionary biology.

The Zhikong scallop *Chlamys farreri* (Jones et Preston, 1904, also known as Chinese scallop) is a subtropical Western Pacific bivalve with wide water-temperature tolerance (−1.5 to 30 °C)^12^ and is naturally distributed along the coasts of Northern China, Korea, Japan, and Eastern Russia. *C. farreri* is epibenthic and semi-sessile. It usually attaches itself to rocks and other hard surfaces with byssal threads, but can detach under adverse conditions and swim away to new habitats^12^. It has an outstanding ability to accumulate PSTs (up to 40,241 μg saxitoxin (STX) eq. per 100 g compared to the 80 μg STX eq. per 100 g safety level for human^13^) and therefore is widely used for studying PST accumulation and transformation^13–15^. *C. farreri* is also a commercially important bivalve with aquaculture production once reaching ~1 million metric tons^16^. It is among the best genetically characterized bivalve species with available linkage, physical and cytogenetic maps^17–21^, fosmid and bacterial artificial chromosome (BAC) libraries^22, 23^, and a large number of expressed sequence tags^24, 25^, making it a good candidate for whole-genome sequencing (WGS).

Here we report the sequencing and analysis of the genome of *C. farreri* along with a comprehensive set of 117 transcriptomes and proteomes covering various organs, development stages, and characteristics of scallop biology. Our multi-omic analyses and associated assays revealed novel genomic features and molecular changes that may underlie aspects of the scallop’s adaptation to semi-sessile and filter-feeding life including the well-developed adductor muscle, sophisticated photoreception system, rapid byssal production, and remarkable resistance to potent neurotoxins.

## Results

### Genome sequencing and characterization

**Fig. 1:**
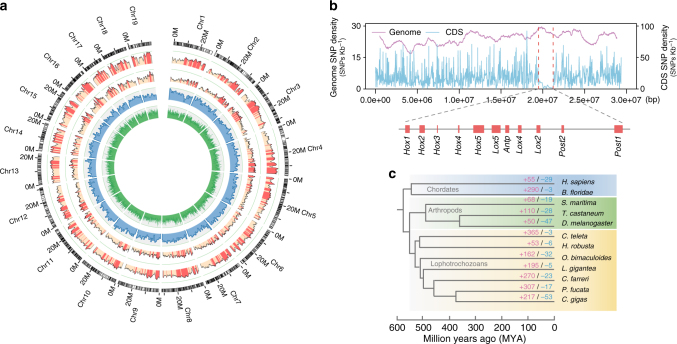
Genome landscape and polymorphism analysis of the scallop *C. farreri*. **a** From outer to inner circles: I, marker distribution on 19 chromosomes at the Mb scale; II and III, single-nucleotide polymorphism (SNP) density across genome (II) or CDS (III) was drawn in 1 Mb sliding windows with a 50 kb step (yellow columns), and polymorphism hotspot regions (500 kb–~5.7 Mb) are colored red; IV and V, gene density and repeat density across the genome, respectively, drawn in 0.1 Mb nonoverlapping windows. **b** SNP density of the *Hox* gene cluster and their genomic background on chromosome 1, showing coding regions of *Hox* genes with extremely low SNP density, compared to high SNP density in the genomic background. The statistical significance for each *Hox* gene is shown in Supplementary Table 12. **c** Gene family expansion/contraction analysis based on 12 representative species. A total of 270 gene families are expanded in the scallop *C. farreri* relative to other bivalves, with the most significant expanded families involved in neurotransmission, immune response, signal transduction, and xenobiotic metabolism (Supplementary Table 13). Expanded and contracted gene families are highlighted in magenta and turquoise, respectively

Genomes of bivalves including *C. farreri* are challenging to sequence and assemble due to their exceptionally high genome heterozygosity^3, 5, 20^. To assemble the highly polymorphic scallop genome, a modified SOAPde novo approach^26^ was used to resolve the complex bubble structures resulting from high genome heterozygosity. Deep sequencing of a 2-year-old *C. farreri* from the Penglai-Red selectively bred population produced 362.8 Gb of clean sequences, with average genome coverage of 382× (Supplementary Table 1). The genome assembly is 779.9 Mb long with a contig N50 size of 21.5 kb and a scaffold N50 size of 602 kb (Supplementary Table 2), and over 80% of the assembly is covered by the longest 1098 scaffolds (>142 kb) (Supplementary Table 3). The assembly statistics of the scallop genome are comparable to or better than those of previously published bivalve genomes (contig N50: 19–38 kb and scaffold N50: 167–804 kb)^3–7^. K-mer analysis (Supplementary Fig. 1) provides an estimate of genome size of ~1 Gb, which is similar to ~1.2 Gb estimated by flow cytometry^23^. The integrity and high quality of the assembly is demonstrated by the mapping of 95.8% of sequencing reads, 97.6–100% of Sanger-sequenced BAC clones, and 99.6–100% of various transcriptome datasets (Supplementary Fig. 2 and Supplementary Tables 4–6), and by the Benchmarking Universal Single-Copy Orthologs (BUSCO)-based completeness assessment (Supplementary Table 7). The assembly was anchored to chromosomes by assigning 949 scaffolds (covering 66.9% of the assembly) to 19 linkage groups (Supplementary Table 8 and Fig. 1a) of a high-density genetic linkage map^20^.

The *C. farreri* genome contains 28,602 protein-coding genes, of which 93.3% encode proteins of at least 100 amino acid residues (aa), and 94.3% are supported by known protein sequences and/or transcriptomic data (Supplementary Fig. 3). Functional analysis via comparison with various public protein databases annotated 24,817 genes, accounting for 86.8% of all the predicted genes (Supplementary Table 9). The *C. farreri* genome contains 261.8 Mb of repetitive sequences accounting for 32.1% of the genome. This percentage is lower than that in most of existing molluscan genomes (Supplementary Table 10). Tandem repeats represent the most abundant repeat type (11.3%), followed by DNA transposons (6.7%), and long interspersed elements (4.4%). Transposable elements (TEs) show higher divergence in *C. farreri* than in other bivalves (Supplementary Figs. 4 and 5), suggesting that they are relatively old.

Polymorphism analysis identified 4.9 million single-nucleotide polymorphisms (SNPs) in the assembled individual (Supplementary Table 11), yielding an intra-individual polymorphism rate of 0.81%. A genome-wide scan of polymorphism based on the assembled and five additionally resequenced individuals identified 108 highly polymorphic genomic regions (≥500 kb) in the genome (Fig. 1a), among which six are longer than 5 Mb (approximately one-fifth of a single chromosome). SNP density in coding sequences (CDSs) varies dramatically among genes, ranging from 0 to ~117 SNPs per kb (Fig. 1a). Particularly, scanning the CDS regions identified a continuous and long SNP-scarce region (~1.74 Mb) on chromosome 1 (Fig. 1b), which harbors an intact cluster of 11 *Hox* genes (3 anterior, 6 central, and 2 posterior): key regulators of bilaterian body plan development^27^. Similarly, low polymorphism was also observed for the *Hox* genes of the scallop *P. yessoensis*, fruit fly, and mouse (Supplementary Fig. 6). The finding of scallop *Hox* genes largely devoid of polymorphism despite high SNP diversity in genomic background (Supplementary Table 12) suggests that the scallop’s body plan formation may be subject to rigid developmental control and its regulators are under strong purifying selection.

Phylogenetic analysis based on 1310 highly conserved orthologous genes (Supplementary Fig. 7) suggests that the scallop lineage diverged from the lineage leading to *Pinctada fucata* and *Crassostrea gigas ~*457 MYA, and Bivalvia diverged from its sister group Gastropoda^28^ ~500 MYA. Gene family analysis (Supplementary Fig. 8) revealed that *C. farreri* has preserved the highest number (7604) of ancestral bilaterian gene families among bivalves. This number is comparable to that in brachiopod *Lingula anatina* (7788), a “living fossil” lophotrochozoan^29^. Compared with other bivalves, 270 gene families are significantly expanded in the scallop lineage (Fig. 1c and Supplementary Data 1) and are predominantly involved in neurotransmission, immune responses, signal transduction, and xenobiotic metabolism (Supplementary Table 13). These expanded gene families are probably important for the scallop’s lineage-specific adaptations and biology. The notable expansion of sodium- and chloride-dependent neurotransmitter transporters in *C. farreri* (61 versus 20–28 in other bivalves; Supplementary Fig. 9) may underlie the scallop’s more developed nervous and vision systems, and higher locomotion activity than other bivalves^9^.

### Muscle regulation and evolution

**Fig. 2:**
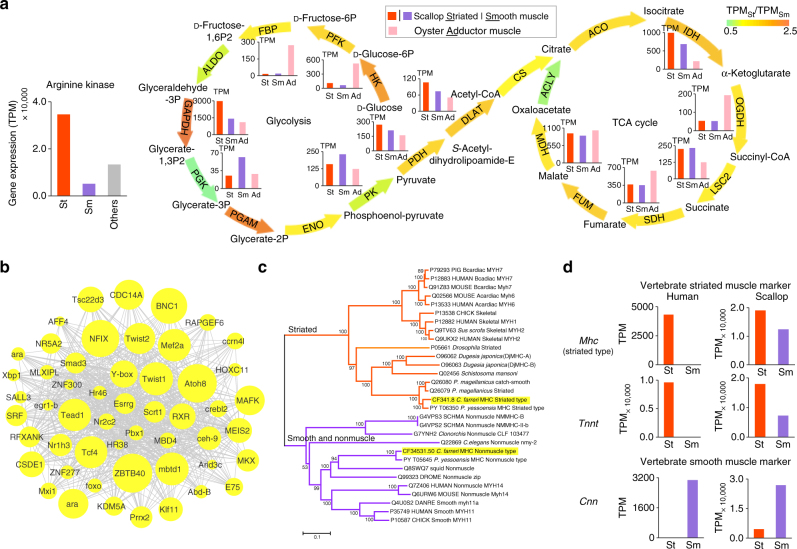
Molecular characterization and evolutionary analysis of scallop adductor muscle. **a** Expression of genes involved in various energy-producing reactions or pathways in striated (ST) and smooth (SM) muscles of *C. farreri* and in the adductor muscle of oyster *C. gigas*, including hydrolysis of phosphoryl arginine, glycolysis, and TCA cycle. The sum of TPM values of genes encoding the same enzyme was defined as the expression level of a given enzyme. Enzymes participating in energy (ATP/energy-rich H^+^) production reactions (GAPDH, PGK, PK, PDH, DLAT, IDH, OGDH, LSC2, SDH, and MDH) generally show higher expression in the scallop *C. farreri* than in the oyster *C. gigas*, whereas the enzymes participating in energy consumption reactions (HK and FBP) show lower expression in *C. farreri* than in *C. gigas* (Supplementary Table 14). Abbreviations of enzymes: HK, hexokinase; PFK, 6-phosphofructokinase; FBP, fructose-1,6-bisphosphatase; ALDO, fructose-bisphosphate aldolase; GAPDH, glyceraldehyde 3-phosphate dehydrogenase; PGK, phosphoglycerate kinase; PGAM, phosphoglycerate mutase; ENO, enolase; PK, pyruvate kinase; OCDH, octopine dehydrogenase; PDH, pyruvate dehydrogenase; DLAT, dihydrolipoamide acetyltransferase; CS, citrate synthase; ACO, aconitate hydratase; IDH, isocitrate dehydrogenase; OGDH, sucA 2-oxoglutarate dehydrogenase; LSC2, succinyl-CoA synthetase β subunit; SDH, succinate dehydrogenase; FUM, fumarate hydratase; MDH, malate dehydrogenase; ACLY, ATP citrate (pro-S)-lyase. **b** The co-expression network of transcription factors (TFs) in the adductor muscle-related module (M3, see Supplementary Data 3). Node size represents the intramodular connectivity of a given gene. **c** Phylogenetic analysis of scallop myosin heavy-chain (*Mhc*) genes. Genes highlighted in yellow are present in the *C. farreri* genome. The scale bar represents 0.1 amino acid substitutions per site. **d** Expression profiles of vertebrate ST- and SM-marker genes in the adductor muscle of *C. farreri*. The human expression data were obtained from the Human Protein Atlas database (http://www.proteinatlas.org/). Expression profiles of striated *Mhc*, *Tnnt*, and *Cnn* are shown and other marker genes (*Mrlc*, *Tnni*,* Ttn*, and *Zasp*) are available in Supplementary Fig. 14

Scallops have a remarkably large adductor muscle (Supplementary Fig. 10) compared to most of sessile and endobenthic bivalves such as oysters, mussels, and clams, probably as adaptation to swimming (Supplementary Movie 1) and the semi-sessile lifestyle. Swimming is an energy-intensive activity, and it is not surprising that the adductor muscle in scallops also serves as the primary organ of energy and glycogen storage and mobilization. We found that arginine kinase, the key enzyme responsible for producing ~ 70% of the ATP needed for phasic contractions (using arginine phosphate as substrate)^30^, shows extremely high levels of transcription in the adductor muscle of *C. farreri*, especially the striated portion (transcripts per million (TPM) = 34,704; ranked sixth among all genes; Fig. 2a). Further analysis of energy-producing pathways (glycolysis, the tricarboxylic acid (TCA) cycle, and oxidative phosphorylation) suggests that most genes related to energy production show higher expression in the *C. farreri*’s striated muscle than in its smooth muscle (Fig. 2a, Supplementary Fig. 11, and Supplementary Table 14), pointing to higher energy dynamics in striated muscle than in smooth muscle. These findings may reflect differences in function, with the large striated muscle responsible for fast, repetitive clapping of valves during swimming and the small smooth muscle responsible for keeping valves closed for long periods at a relatively low energy cost^8^. Interestingly, enzymes participating in energy (ATP/energy-rich H^+^) production (e.g., glyceraldehyde 3-phosphate dehydrogenase, pyruvate dehydrogenase, dihydrolipoamide acetyltransferase, isocitrate dehydrogenase, succinyl-CoA synthetase β-subunit in glycolysis and in the TCA cycle) generally show higher expression in the scallop *C. farreri* than in the oyster *C. gigas*, but the reverse is true for the enzymes related to energy consumption (HK and FBP; Fig. 2a and Supplementary Table 14). This finding may reflect adaptations to lifestyles with different levels of energy demand: high in semi-sessile scallop and low in sessile oyster.

To understand transcriptomic regulation in the adductor muscle, we constructed gene co-expression networks from 35 adult transcriptome datasets, and identified M3 as the only adductor muscle-related module (significantly enriched in both striated muscle- and smooth muscle-related genes; Supplementary Data 2 and Supplementary Fig. 12). Analysis of transcription factors (TFs) in M3 suggests that *Twist*, *Nfix* and *Zbtb40* are among the top-ranked TFs with the highest intramodular connectivity (Fig. 2b and Supplementary Data 3), with the former two known as key TFs in animal myogenesis^31, 32^. *Twist*, the master regulator of myogenesis, loses expression in adult muscles of *Drosophila* ^31, 33^ and shows high expression in the adductor muscle of the adult scallop (Supplementary Fig. 13), possibly related to the different modes of adult muscle growth: determinate for *Drosophila* ^34^ and indeterminate for the scallop^35^.

It is also interesting that key marker genes^36^ that distinguish vertebrate striated muscles (striated *Mhc* (myosin heavy chain), *Tnnt, Tnni*, *Ttn*, and *Zasp*) from smooth muscles (*Cnn*) show high expression in both striated and smooth muscles of *C. farreri* (Fig. 2c, d, Supplementary Fig. 14, and Supplementary Table 15), revealing the “hybrid” nature of scallop striated and smooth muscles that are not as distinctive as in vertebrates. The expression of the same fast contractility components in smooth as well as striated muscle has been reported for other scallop species^37, 38^, ascidians^39, 40^, and flatworms^41–43^. Our findings together with those from other studies suggest that smooth and striated muscles in at least some invertebrates are not as differentiated as in vertebrates, thereby probably representing a plesiomorphic state, and still use shared basic building blocks (i.e., gene components) but in different organizations^41^.

### Opsin diversity and retina evolution

**Fig. 3:**
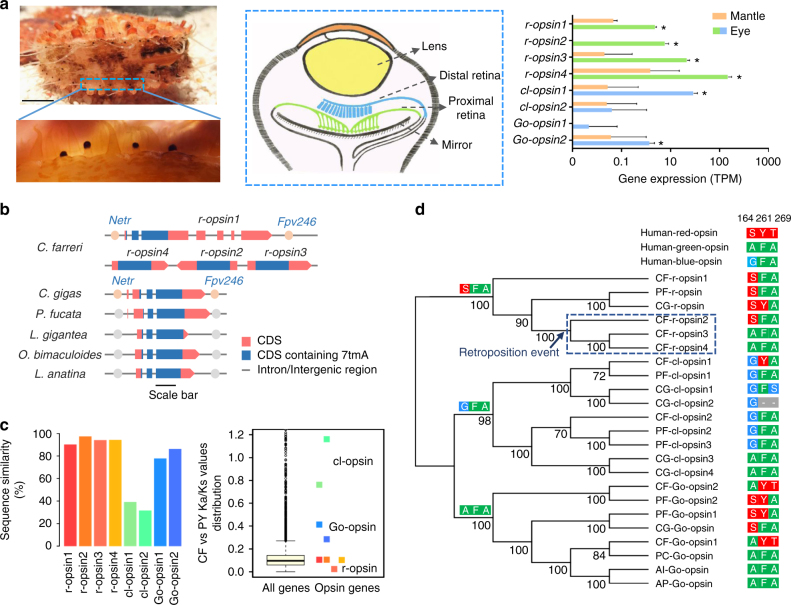
The evolution of opsin diversity and photoreception tuning in *C. farreri*. **a** Morphology of scallop eyes scattered along the edge of mantles (left, scale bar: 1 cm), schematic structure of a typical scallop eye (middle), and expression of diverse opsin genes in scallop eyes (right). Eye samples from three individuals were used in expression evaluation with standard error shown for eye and mantle groups. The asterisks indicate genes showing significantly higher expression in eyes than in the mantle (*p*-value < 0.05, the exact test by edgeR). **b** R-opsin gene structures of Mollusca and Branchiopoda. Exons rather than introns were plotted in proportion, with scale bar representing 500 bp. NETR (neurotrypsin) and FPV246 (putative ankyrin repeat protein) are the conserved neighboring genes. **c** Sequence similarity and Ka/Ks values of all opsin genes between the scallops of *C. farreri* (CF) and *P. yessoensis* (PY). These data were calculated based on full protein sequences. The black line insides the box indicates the median value, and the whiskers extend from the first or third quartiles to the minimum or maximum values. **d** Bivalve opsin phylogeny and variation at key functional sites sensitive to various light ranges. Species abbreviations: *Chlamys farreri* (CF), *Pinctada fucata* (PF), *Crassostrea gigas* (CG), *Argopecten irradians* (AI), *Patinopecten caurinus* (PC) and *Argopecten purpuratus* (AP). Site combinations of “SFA,” “GFA” and “AFA” above the major branches are the putative ancestral bivalve types deduced from extant species. Colors of the sites correspond to the colors or wavelengths of opsin sensitivity in human opsins

Scallops possess a large number of sophisticated non-cephalic eyes along the edge of their mantle (Fig. 3a) and thus are thought to have the best vision system among bivalve molluscs^44^. Opsins of the G-protein-coupled receptor (GPCR) family are key light-sensing proteins responsible for visual signal transduction^45^. On the basis of sequence alignment with known opsins and GPCR-domain searches, we identified eight vision-related opsin genes in the scallop genome, including four *r-opsin*, two *Go-opsin* and two c-like-opsin (*cl-opsin*) genes (Supplementary Fig. 15). As the characteristic opsin type in invertebrates^46^, *r-opsin* (also known as Gq-coupled opsin) genes are significantly expanded (four copies) in the *C. farreri* genome, compared to a single gene copy found in other molluscs (Fig. 3b). In *C. farreri*, *r-opsin1* is likely the ancestral copy, because it shows relatively conserved gene structure and neighboring genes with the single-copy *r-opsin* genes in other molluscs (Fig. 3b). Interestingly, *r-opsin2, r-opsin3*, and *r-opsin4* are all intronless (Fig. 3b) and are likely retrogenes generated via retroposition of a messenger RNA (mRNA) transcript^47^. Phylogenetic analysis suggests that these intronless r-opsins were generated by stepwise duplications from the original intron-containing gene (i.e., *r-opsin1*), with *r-opsin2* generated by retroposition first, followed by tandem duplications that produced *r-opsin3* and *r-opsin4* (Fig. 3d). The latter, *r-opsin4*, is the favored gene copy in the eyes of *C. farreri*, because its expression (average TPM = 2415.0) greatly exceeds that (average TPM = 2.3–46.4) of other r-opsins (Fig. 3a). Intronless genes are more efficient to transcribe as no post-transcriptional splicing is needed. For example, approximately 70% of early zygotic genes of *Drosophila* are intronless^48^, due to a need for efficient transcription during rapid cell divisions in early development^48, 49^. The utilization of intronless opsins in *C. farreri* may represent an adaptive change for enhancement of transcription efficiency in support of the scallop’s unusual and advanced multi-eye visual system.

Scallop eyes possess a unique double-layered retina (Fig. 3a), which are equipped with different photoreceptors sensitive to light of different wavelengths and play different roles in visual behaviors^50^. The proximal retina consists of rhabdomeric photoreceptor cells (also found in most of invertebrate eyes), whereas the distal retina consists of ciliary photoreceptor cells (similar to rod and cone cells of vertebrates)^51^. Key genes participating in rhabdomeric and ciliary phototransduction pathways were identified here in the *C. farreri* genome, and higher gene expression of the Gq-coupled rhabdomeric pathway implies that the proximal retina plays prominent roles in the scallop visual system (Supplementary Table 16). The evolutionary origins of the two layers of the retina remain enigmatic. It has been suggested that the distal retina might have evolved later than the proximal retina^51, 52^ and thus is likely under relaxed selection pressure. This hypothesis is, to some extent, supported by our analysis of opsin genes from two scallop species (*C. farreri* and *P. yessoensis*): ciliary opsins expressed in the distal retina showed significantly lower sequence conservation (*t*-test *p*-value = 0.04), greater Ka/Ks values (*t*-test *p*-value = 0.03), and much weaker transcription relative to rhabdomeric opsins expressed in the proximal retina (Fig. 3a, c). The presence (addition or retention) of the otherwise vertebrate-specific distal retina may represent an evolutionary innovation giving the scallop the potential to form image and detect movement^53^.

Scallop eyes can detect a wide spectrum of light wavelengths (*λ*_max_ = 480–540 nm^54^), with the proximal retina sensitive to short wavelengths, whereas the distal retina to long wavelengths^50^. Various studies have shown that three key amino acid positions in opsins (164, 261, and 269 in reference to rhodopsin of *Bos taurus* ^55^) are crucial determinants of spectral sensitivity to short or long wavelengths^56^. *R-opsin3* and *r-opsin4*, the two opsins derived from gene duplication and most highly expressed in the proximal retina of *C. farreri*, have “AFA” at these sites (just as human green opsin does; Fig. 3d), adding a potentially valuable variant to the ancestral “SFA” type found in the scallop (*r-opsin1* and *r-opsin2*) and other bivalves. Apparently, the gene duplication increased the diversity of the r-opsin protein at core functional sites in its amino acid sequence; this change may broaden the scallop’s spectral sensitivity. Similarly, new functional site variants of Go-opsin (AYT) and cl-opsin (GYA) were observed in *C. farreri* (Fig. 3d) that may allow the scallop to sense different ranges of long-wavelength light because SYT in humans corresponds to red-light sensitivity^56^. These results show that scallop eyes are not only numerous and structurally advanced but also equipped with a diverse set of opsins including novel intronless genes resulting from retroposition and tandem duplication, and new opsin variants that are polymorphic at functional sites in amino acid sequence and may broaden spectral sensitivity. These notable molecular features may provide scallops with enhanced vision or light sensitivity as part of their adaptation to epibenthic semi-sessile life.

### Byssal proteins and secretion regulation

**Fig. 4:**
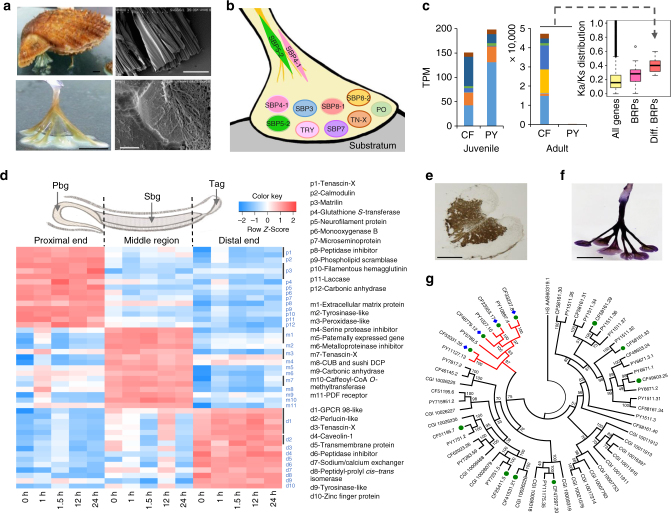
Scallop byssal protein composition and molecular regulation of byssal secretion. **a** The morphology and microstructures of the byssal threads of *C. farreri*. Scale bars are 5 mm (top-left and bottom-left), 0.5 mm (top-right), and 2. 5μm (bottom-right). The full set of microstructure images across the whole byssus is presented in Supplementary Fig. 16. **b** Schematic illustration of byssal protein composition according to mass spectrometric analysis. TN-X, tenascin-X; TYR, tyrosinase; PO, peroxidase; SBP, scallop byssal proteins identified by Miao et al.^62^. **c** Gene expression and the Ka/Ks substitution rate of foot-specific byssal proteins. Expression was compared between *C. farreri* (CF) and *P. yessoensis* (PY, a species without adult byssal secretion) in the juvenile and adult foot. The Ka/Ks ratio was calculated based on homologous gene pairs from CF and PY for all proteins, all BRPs and the BRPs differentially expressed between the adult feet of CF and PY (Diff. BRPs). The black line inside the box indicates the median value, and the whiskers extend from the first or third quartiles to the minimum or maximum values. The color code correspondence is detailed in Supplementary Table 17. **d** Spatial and temporal expression of top 15 genes that are highly and differentially expressed in proximal, middle, and distal regions of the *C. farreri* foot. The sampling time points refer to the time after removal of old byssal threads. Genes from the same gene family are grouped together (represented by a bar). Abbreviations: Pbg, primary byssal gland; Sbg, secondary byssal gland; Tag, tip attachment gland. **e** A catechol oxidase activity assay in cryosectioned foot glands reveals tyrosinase activity on l-tyrosine as a substrate. **f** Nitroblue tetrazolium staining of byssal threads reveals abundant presence of DOPA. Scale bars represent 1 mm **e** and 5 mm **f**. **g** The maximum likelihood-based phylogeny of bivalve tyrosinases (*Tyr*). The *Tyr* genes expressed in juvenile and/or adult foot (TPM ≥ 2) are indicated by green dots. Those specifically expressed in adult foot (fold change_[foot/ave_nonfoot_organ]_ = 206–1729) are indicated by blue diamonds, showing closer phylogenetic relationships (in the red cluster). Numbers above the branches are bootstrap values from 1000 replicates. CGI: *C. gigas*

Many aquatic animals including bivalves, barnacles, and sandcastle worms have evolved effective strategies for adhesion as adaptation to turbulent currents^57^. As an ancestral feature of bivalves^58^, byssal attachment is an essential part of the sessile or semi-sessile lifestyle characteristic of diverse bivalve families, but, to date, has been extensively studied only in mussels^59–61^. In contrast to mussels, scallop byssal attachment is characterized by abundant secretion of byssal proteins and temporary attachment (in concert with swimming behavior; Fig. 4a). The scallop byssus has different ultrastructure and morphology in different regions (Fig. 4a and Supplementary Fig. 16), indicating compositional and mechanical complexity. Mass spectrometric analysis identified 16 candidate byssus-related proteins (BRPs) in the whole byssal adhesive plaques of *C. farreri* (Supplementary Figs. 17 and 18 and Supplementary Data 4), including seven previously identified scallop byssal proteins (SBPs^62^). Functional annotation of these BRPs suggests that they potentially involve in oxidative reactions (tyrosinase and peroxidase), extracellular matrix consolidation (tenascin-X), and anti-biodegradation (serine protease inhibitor and metalloproteinase inhibitor) (Fig. 4b, Supplementary Data 4, and Supplementary Fig. 19). Of the identified scallop BRPs, none shows protein similarity to a well-known set of 11 mussel BRPs^63^, but up to eight to an expanded set of 48 mussel BRPs recently identified by Qin et al.^64^ (Supplementary Data 4). Four scallop BRPs (CF48907.12, CF47691.7, CF44339.32, and CF30077.9) that do not have either protein/domain annotations or similarity to mussel BRPs likely represent novel SBPs. Of the 16 identified BRPs, 12 show high and specific expression in the foot of *C. farreri* but nearly no expression in the foot of the adult Yesso scallop *P. yessoensis*, a species that is free living and does not produce byssi in adulthood (Fig. 4c and Supplementary Table 17). Ka/Ks analysis indicates that these BRPs diverged more rapidly than other BRPs did (Fig. 4c), reflecting differential selection in the two scallop species possibly because of different requirements for byssal attachment at the adult stage.

Byssal secretion is a complicated process involving a series of biochemical reactions occurring in various byssal and enzymatic glands located in different regions of the foot^65^. To investigate the molecular mechanism of byssal secretion, we conducted comprehensive temporal and spatial gene expression profiling by sequencing 45 transcriptomes of three foot regions (proximal, middle, and distal) and at five time points during byssal secretion. Our results show that the three foot regions have different gene expression patterns, reflecting their different roles during byssogenesis (Fig. 4d). The proximal end of the foot, where the primary byssal gland resides and byssal ribbons are secreted, is characterized by high expression levels of connective proteins tenascin-X and matrilin and various related enzymes. The middle foot region where the secondary byssal and/or enzymic glands are located and the byssal ribbon sheath or envelope is formed, predominantly shows expression of a variety of tyrosinases (Fig. 4g): enzymes crucial for mussel byssogenesis that catalyze the formation of a strong adhesive, 3,4-dihydroxyphenylalanine (DOPA)^66^. Tyrosinases’ participation in scallop byssogenesis is supported by the enzymatic activity in foot glands and high abundance of DOPA in byssi (Fig. 4e, f). The distal end of the foot shows dramatically increased transcription of signal transduction-related GPCR98-like proteins^67, 68^ at the initial stage (<1 h) of byssal secretion, and likely plays a role in guiding the search for suitable spots for attachment.

### Neurotoxin accumulation and transformation

**Fig. 5:**
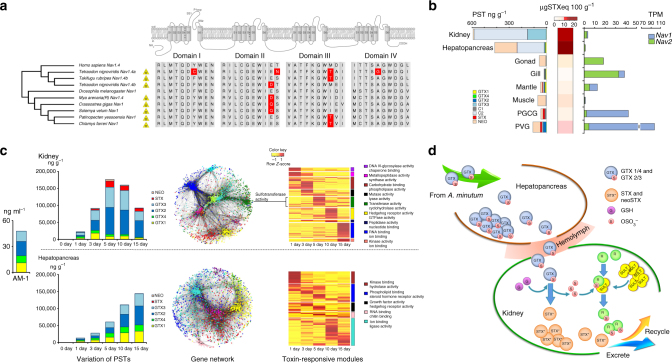
Toxin resistance and response regulatory networks in the scallop kidney and hepatopancreas. **a** Amino acids conferring PST or TTX resistance on sodium channel *Nav1* (highlighted in red) identified in this and other studies^69, 75–79^. Skull signs indicate toxin-producing and -resistant species. **b** Expression of sodium channels *Nav1* and *Nav2*, and PST concentration and toxicity levels in major organs of *C. farreri*. Toxicity (μg STX eq. per 100 g) was determined by converting total concentration of PSTs to micrograms of STX equivalents per 100 g of tissue^80^. **c** Temporal variations in abundance of different PSTs and gene networks in the kidney and hepatopancreas after exposure to the toxic alga *Alexandrium minutum*. Toxin response modules were identified by enrichment analysis of DEGs during exposure to *A. minutum* and each module was annotated with the two most significantly enriched GO term(s). The green module is the largest kidney-specific response module, where cytosolic sulfotransferase (*Sult*) genes are enriched (Supplementary Data 10) and highly expressed on day 5 after *A. minutum* exposure (as indicated in the heat map). **d** A schematic diagram showing different roles of the scallop hepatopancreas and kidney in toxin metabolism, with the hepatopancreas primarily responsible for PST accumulation, whereas the kidney primarily for PST transformation mediated by SULTs

Bivalves can tolerate and accumulate potent neurotoxins such as PSTs, although the molecular mechanism of toxin resistance in bivalves is not well understood. Like tetrodotoxin (TTX) of puffer fish, PSTs attack the nervous system by blocking sodium channels on nerve cell membranes and by inhibiting transduction of an action potential^69^. We identified two sodium channel genes, *Nav1* and *Nav2*, in the *C. farreri* genome. *Nav1* is the primary sodium channel in animal nervous systems^70^ and is targeted by PSTs. We found that the scallop’s *Nav1* has a potentially toxin-resistant T mutation at position 1425 (in reference to rat sodium channel IIA^69^; Fig. 5a): the corresponding mutation in rat *Nav1* yields a 15-fold increase in resistance to STX (the most potent PST) and a 15-fold increase in resistance to TTX^71^. This mutation is also present in the *Nav1* genes of two puffer fish species^72, 73^, *Tetraodon nigroviridis* and *Takifugu rubripes* (Fig. 5a), which have strong toxin resistance, pointing to convergent evolution of toxin resistance in the scallop and puffer fish. Furthermore, our analysis revealed a Q mutation at position 945 in *C. gigas* and Atlantic awning clam *Solemya velum* (Fig. 5a), which has been shown to increase STX resistance up to 19,880-fold in a rat sodium channel^73^. The presence of these two novel mutations that are known to increase toxin resistance in other organisms may explain bivalves’ amazing ability to tolerate neurotoxins. Moreover, gene expression analysis in combination with PST quantification showed that toxin-rich organs (hepatopancreas and kidney) are largely devoid of sodium channel gene expression (Fig. 5b and Supplementary Table 18), which may represent another novel adaptation of the scallop for tolerance of high concentrations of PSTs via down-regulation of their targets. This situation is consistent with the hepatopancreas and kidney being the most toxin-rich or toxin-tolerant organs in the scallop.

Accumulation and transformation of PSTs in bivalves are well documented^13, 15^, but detailed processes and mechanisms remain obscure. To gain a deeper understanding of PST accumulation and transformation in scallops, we comprehensively studied PST accumulation and transformation in *C. farreri* by qualitatively and quantitatively analyzing a variety of PSTs in six scallop organs across five time points after exposure to PST-producing microalgae *Alexandrium minutum*. We found that the hepatopancreas and kidney are the two organs with the highest concentrations of PSTs, but the kidney is more toxic than hepatopancreas (Fig. 5b). This is a new and significant finding because the kidney has been ignored in previous PST studies. The hepatopancreas maintains a similar PST profile as the input microalgae over time; however, the PST profile of the kidney gradually changes with time and the most dramatic toxin change (from input GTXs to highly toxic STX; Supplementary Fig. 20) takes place after 5 days of *A. minutum* exposure (Fig. 5c, Supplementary Fig. 21, and Supplementary Datas 5 and 6). This finding suggests that the scallop hepatopancreas and kidney function differently, with the former mostly accumulating the incoming toxins, whereas the latter in toxin transforming and/or eliminating them. To study the molecular mechanisms of PST accumulation and transformation, we sequenced 36 transcriptomes (Supplementary Table 19) of the hepatopancreas and kidney after *A. minutum* exposure (across six time points, each represented by three individuals) and constructed a gene co-expression network for both organs (Supplementary Fig. 22). Nine and five modules were identified as toxin-responsive (TR) modules in the kidney and hepatopancreas, respectively (Fig. 5c and Supplementary Data 7). Kidney TR modules were found to be involved in diverse molecular functions (e.g., RNA/ion/carbohydrate binding, transferase activity, peptidase activity, and kinase activity), whereas hepatopancreas TR modules primarily participated in a variety of “binding” activities (Supplementary Datas 8 and 9). Notably, genes in the green module were highly expressed on day 5 after *A. minutum* exposure, coinciding with the highest transformation of PSTs in the kidney (Fig. 5c). The green module is significantly enriched with cytosolic sulfotransferase (*Sult*) genes (enrichment *p*-value = 5.9e−3; Fig. 5c and Supplementary Data 8), which may mediate the transfer of a sulfate group from a donor molecule (such as GTXs) to various acceptor molecules, endogenous metabolites, and xenobiotics^74^. The *Sult* family is significantly expanded in the *C. farreri* genome (83 genes versus 26 in the oyster, 31 in the pearl oyster, 13 in humans, and 8 in the fly), and the *Sult* genes showing significant up-regulation in the kidney during *A. minutum* exposure all belong to the clade that is expanded in *C. farreri* or bivalves (Supplementary Fig. 23 and Supplementary Data 10). Collectively, our results suggest that the scallop hepatopancreas and kidney act as two major “centers” for toxin accumulation and transformation, respectively. The expanded *Sult* genes likely participate in conversion of GTXs to more toxic STX (Fig. 5d), which may give the scallop a powerful deterrent against predation, while the novel mutations in *Nav1* revealed in this study may provide scallops and other bivalves with the ability to tolerate those neurotoxins. It seems that dinoflagellates produce neurotoxins to inhibit grazing by filter feeders, but bivalve molluscs have adopted novel sodium channel variants to tolerate neurotoxins and converted the toxins to even more toxic forms for their own defense against predation. Our findings highlight how simple mutations and expansion in one or two key genes may have profound implications for an organism’s adaptation to the environment and the complex interactions with other organisms.

## Discussion

Bivalves are a fascinating group of animals, which, despite long evolutionary history dating back to the early-Cambrian, are still abundant and thriving as highly successful filter feeders dominating diverse benthic environments. Their remarkable adaptation to benthic life is not well studied at genomic levels. We sequenced the genome of the Zhikong scallop and collected extensive transcriptomes and proteomes to study molecular or genomic changes related to several of its adaptive features. Our analyses identified significant expansion in 270 gene families that may be important for *C. farreri*’s biology and adaptation. The scallop’s large striated muscle shows heightened energy dynamics and is not fully differentiated from its smooth muscle. The sophisticated noncephalic multiple eyes of *C. farreri* are supported by the predominant use of novel intronless *r-opsin* genes (derived from retroposition and tandem duplication) and by diverse opsin variants for possibly broadened spectral sensitivity. The rapid secretion of byssi is enabled by a spatially differentiated foot and multiple proteins/enzymes including the expanded family of tyrosinases. The scallop uses the hepatopancreas to accumulate algae-derived neurotoxins and uses the kidney to transform them into highly toxic compounds by means of the expanded family of sulfotransferases, probably as deterrence against predation, while its own remarkable resistance to neurotoxins may be explained by mutational and expressional modulation of sodium channels. These molecular innovations may be important for the scallop’s semi-sessile lifestyle as a filter feeder, suggesting that simple expansion and mutation of a few key genes may have profound effects on an organism’s phenotype and adaptation.

## Methods

Brief description of methods. The whole genome of a 2-year-old *C. farreri* was sequenced using the Illumina HiSeq 2000 platform through the construction and sequencing of both short-insert (180, 300 and 500 bp) and long-insert (2, 5, 10, 20 and 30 kb) DNA libraries. The genome size of *C. farreri* was estimated based on the 19-mer frequency distribution. To address the problem of high genome heterozygosity, a hierarchical strategy based on a modified version of SOAPdenovo^4^ was used for the assembly of the *C. farreri* genome. The integrity of the final assembly was assessed by means of four data sets: four BAC sequences, WGS data, transcriptome data, and an 843-BUSCO metazoan subset of genes. The assembly was further anchored to chromosomes based on a high-density genetic linkage map^10^, through the assignment of the scaffolds to 19 linkage groups. For repeat annotation, tandem repeats were predicted using the software Tandem Repeats Finder^11^, and TEs were predicted via two approaches (homology-based method and de novo prediction). To predict genes in the *C. farreri* genome, three approaches (homolog-based, de novo, and transcriptome-based predictions) were employed. Functional annotation of the protein-coding genes of *C. farreri* was performed by searching the SwissProt, TrEMBL, InterPro, GO (gene ontology), and KEGG (Kyoto Encyclopedia of Genes and Genomes) databases. Thirteen adult tissues/organs of the scallop were chosen for transcriptome sequencing, including striated muscle, smooth muscle, foot, hepatopancreas, kidney, female gonad, male gonad, gill, eyes, mantle, cerebral ganglion, and visceral ganglion. Differentially expressed gene (DEG) analysis was carried out using edgeR^23^ with three biological replicates, and genes with a fold-change value ≥2 and adjusted *p*-value <0.05 were defined as significant DEGs. To characterize the polymorphism in the *C. farreri* genome, reads from the sequenced individual and five additional resequenced individuals were aligned to the assembled genome for SNP calling using the BWA^6^ software. Genomic regions or CDSs with high SNP density subjected to one-sided Fisher’s exact test by comparing to the corresponding chromosomal background, and the distribution of SNP density among chromosomes or genes was visualized using the Circos software^26^. The OrthoMCL pipeline^27^ was used to define gene families for the selected species. For phylogenetic analysis, we selected orthologous genes using a tree-based approach PhyloTreePruner^28^, and the phylogenetic tree was constructed using RAxML^29^. To estimate the divergence time for *C. farreri* and other metazoans, the first and second codon positions of the orthologs were extracted for Bayesian dating using the MCMCtree program implemented in PAML^31^, with reference divergence time of selected species retrieved from the TimeTree^33^ database. The evolutionary dynamics (expansion/contraction) of gene families were analyzed in the software CAFÉ^34^, and GO enrichment analysis was performed using the EnrichPipeline^35^. For muscle analysis, we compared the transcript abundance of various enzymes involved in glycolysis, TCA cycle, and oxidative phosphorylation pathways between different types of scallop muscles or between scallop and oyster muscles. Co-expression gene networks were constructed by means of WGCNA^36^ using 35 transcriptomes from adult tissues/organs, and module enrichment of muscle-overrepresented genes was conducted by the hypergeometric test (*p* < 0.05). The expression profile of vertebrate muscle marker genes^38^ in the scallop was determined using the average TPM value of three biological replicates, and the corresponding value in human was obtained from the HPA dataset (http://www.proteinatlas.org/). Putative opsin genes in the scallop and other bivalves were identified by BLAST-based searching against known opsin genes of other animal species at an e-value threshold of 1e−5, and only those containing seven transmembrane domains and the lysine site (296 K) were kept for subsequent analysis. The opsin phylogeny was constructed by the Bayesian method^39^ using the sequences of seven transmembrane domains. Ka/Ks values were estimated by means of Ka_Ks_calculator 2.0^40^ using the YN method. Key genes involved in rhabdomeric and ciliary phototransduction pathways were identified by homology-based search against the known genes from *Homo* and *Drosophila*, and putative light sensitivity of bivalve opsin genes was determined by means of amino acid combinations at key positions (164, 261 and 269). The whole protein sample as well as major sodium dodecyl sulfate-polyacrylamide gel electrophoresis fractions extracted from byssal adhesive plaques by using the method of Miao et al.^43^ were subject to mass spectrometric analysis. The mass spectrometry raw data were searched against the predicted proteins from the *C. farreri* genome using Mascot v.2.3.0. To be stringent, the identified proteins with ≤ 1 unique matching peptide in both datasets and with expression ratio_[foot/ave_nonfoot_organ]_ ≤ 2 were excluded from further analysis. Functional annotation of scallop candidate BRPs was performed by searching against SwissProt, Pfam, InterPro, SMART, and SignalP databases. Microstructures of the byssal thread were examined by scanning electron microscopy (Hitachi S-3400N). Forty-five RNA-seq libraries covering three foot regions (proximal, middle, and distal) and five time points after the removal of byssal threads (0, 1, 1.5, 12 and 24 h) were subject to Illumina sequencing and the overrepresented genes in each foot region were identified by DEG analysis using the edgeR package^23^. A nitroblue tetrazolium staining assay was performed on the whole byssal threads, and a catechol oxidase assay for in situ detection of tyrosinase activity. For phylogenetic analysis of tyrosinases, a maximum likelihood (ML) tree was constructed using RAxML^29^ and the robustness of the tree was tested by reanalysis of 1000 bootstrap replicates. The voltage-gated sodium channel protein (*Nav*) sequences of *C. farreri* and other bivalves were identified via homology-based searches with an e-value threshold of 1e−10. Amino acids positions putatively conferring PST and TTX resistance were identified based on conservation of previously reported sites^45–50^. Thirty-six RNA-seq libraries of the hepatopancreas and kidney from scallops fed with toxic *A. minutum* were subject to Illumina sequencing, and DEGs were identified using R package edgeR^23^. The co-expression gene networks for the hepatopancreas and kidney were constructed using the R package WGCNA^36^, and over-representation analysis of the TR genes was performed for each module by a hypergeometric test (*p* < 0.05) to identify TR modules. GO enrichment analysis of each TR module in the networks was conducted using the EnrichPipeline^35^. The cytosolic sulfotransferase (*Sult*) genes were identified in the genomes of three bivalves, *H. sapiens* and *D. melanogaster* using BLAST with an e-value threshold of 1e−5. The ML tree of SULTs was constructed using RAxML^29^ and the robustness of the tree was tested by reanalysis of 1000 bootstrap replicates. More detailed description of the above methods can be found in the Supplementary Information.

### Data availability

This genome project has been registered in NCBI under the BioProject accession PRJNA185465. The sequencing data of *C. farreri* have been deposited in NCBI Sequence Read Archive under the accession numbers of SRX1305705, SRX2486272, SRX2486273, SRX2486281, SRX2486284, SRX2486300, and SRX2913253-SRX2913260 for genomic data; and SRX2444844-SRX2444876, SRX2508197-SRX2508199, SRX2444668-SRX2444682, SRX2444950-SRX2444979 and SRX2445405-SRX2445440 for transcriptomic data. The proteomic data have been deposited in PRIDE Archive database under the accession numbers PXD007932 and PXD007987. The *C. farreri* genome assemblies (including an updated version improved by the addition of ~ 26 Gb PacBio data), gene sequences, and annotation data are available at the scallop genome website (http://mgb.ouc.edu.cn/cfbase/html/).

## Electronic supplementary material

Supplementary Information

Description of Additional Supplementary Files

Supplementary Movie 1

Supplementary Data 1

Supplementary Data 2

Supplementary Data 3

Supplementary Data 4

Supplementary Data 5

Supplementary Data 6

Supplementary Data 7

Supplementary Data 8

Supplementary Data 9

Supplementary Data 10
