# Supplementary material for: Scallop genome reveals molecular adaptations to semi-sessile life and neurotoxins
Source: Nat Commun. 2017 Nov 23;8:1721. doi: 10.1038/s41467-017-01927-0 (PMC5700196; doi:10.1038/s41467-017-01927-0)
Supplement: Supplementary file 1 — Supplementary Information [file 41467_2017_1927_MOESM1_ESM.pdf]

Supplementary Methods

Genome sequencing and estimation of genome size

Genomic DNA of a two-year-old *C. farreri* was obtained by the conventional phenol/chloroform extraction method[1]. Short-insert (180, 300, and 500 bp) paired-end libraries were generated using the Next Ultra DNA Library Prep Kit for Illumina (NEB, USA) following manufacturer's recommendations. Large-insert (2, 5, 10, 20, and 30 kb) mate-pair libraries were prepared following the Cre-lox recombination-based protocol[2]. All DNA libraries were sequenced on the Illumina HiSeq 2000 platform. After filtering out the adapter sequences and low-quality and duplicated reads, a total of 362.78 Gb of sequencing data were obtained (382-fold coverage of the genome) and were used for genome assembly.

The genome size of *C. farreri* was estimated based on the 19-mer frequency distribution using the following formula[3]: Genome size = (total number of 19-mers)/ (position of peak depth). The genome size estimated by the 19-mer analysis was used for calculating the statistical parameters of the assembly.

Genome assembly

To solve the problem of high polymorphism, a modified version of SOAPdenovo[4] was used for scallop genome assembly. Briefly, for assembly of heterozygous contigs, contigs with depth less than 87 were collected at the following SOAPdenovo settings: -e 1 -M 0 -R. Then, WGS reads were mapped back to the heterozygous contigs by SOAPdenovo to generate links, and a minimum of three read pairs were necessary to define an effective link. The links containing orientation and distance information between heterozygous contigs were used to cluster and classify heterozygous contigs. In a bubble structure, two contigs represented two potential haplotypes, and the longer one was retained in the final assembly to maximize the integrity of assembly.

For the scaffolding assembly, all short-insert paired-end reads and large-insert mate-pair reads were mapped onto contig sequences using SOAPdenovo. Contigs located in repetitive sequences with high similarity usually have high coverage and are prone to conflicting connections, which were masked during scaffolding. Then, a

hierarchical strategy was applied to generate compatible connections for scaffolding of contigs by adding data from short-insert reads to long-insert reads step by step. Finally, all short-insert reads were realigned back onto the scaffold sequences to fill intra-scaffold gaps and thus to obtain the final genome assembly.

Quality assessment of the genome assembly

Integrity of the final assembly was assessed by means of four data sets: four BAC sequences, WGS data, muscle transcriptome data, and an 843-BUSCO metazoan subset of genes. BAC sequences were aligned to the genome assembly using LASTZ[5] with parameters M=254 K=4500 L=3000 Y=15000 --seed=match12 --step=20 --identity=85. The WGS reads were aligned onto the final assembly using Burrows-Wheeler Aligner[6] with settings "-n 15 –o 1 –e 10" considering high polymorphism[4]. The muscle transcriptomes *de novo* assembled by Trinity[7] were mapped to the genome assembly using BLAT with default parameters and an identity cutoff of 80%. BUSCO[8] completeness assessment was conducted using default parameters and 843 metazoan single-copy orthologs.

Linkage map-based chromosome anchoring

A total of 3,806 2b-RAD[9] marker sequences were obtained from a high-density linkage map of *C. farreri*[10] and aligned back to the assembly using BLAST at the following settings: -e 1e-4 -F F -G 5 -E 2 -W 7 -r 2 -q -3 -m 8. Only markers with a unique location were used for anchoring and orienting scaffolds to different linkage groups. Scaffolds in conflict with the genetic map (such as markers from a different linkage on the same scaffold) were checked manually with 10 kb mate-paired reads, and nine scaffolds were broken apart at the point of low coverage by mate-paired reads.

Genome annotation

For repeat annotation, tandem repeats were predicted using software Tandem Repeats Finder (TRF)[11], and TEs were predicted via two approaches (homology-based method and *de novo* prediction). RepeatMasker[12] and RepeatProteinMask were used for

homology-based TE prediction: we ran searches against RepBase sequences with default parameters. *De novo* TEs in the genome were detected by RepeatMasker based on a *de novo* repeat library, constructed by RepeatModeller (http://www.repeatmasker.org/RepeatModeler.html). Repeat sequences from other lophotrochozoan genomes (*L. anatina*, *Octopus bimaculoides*, *Lottia gigantea*, *P. fucata* and *C. gigas*) were annotated using the same pipeline.

To predict genes in the *C. farreri* genome, three approaches (homolog-based, *de novo* and transcriptome-based predictions) were employed. Protein sequences from eight sequenced genomes (*Amphimedon queenslandica*, *Strongylocentrotus purpuratus*, *Branchiostoma floridae*, *Capitella teleta*, *Helobdella robusta*, *L. gigantea*, *C. gigas* and *P. yessoensis*) were downloaded from Ensembl (Release 70) or JGI. Protein sequences of the selected species were aligned to the repeat-masked scallop genome using TBLASTN with an E-value cutoff of 1e-5. To obtain accurate spliced alignments, homologous genome sequences were aligned with the matching proteins using Genewise[13]. For *de novo* predictions, we used three *ab initio* gene prediction software tools—Augustus[14] (version 2.5.5), GlimmerHMM[15] (version 3.0.1), and SNAP[16]—to predict coding genes. High-throughput massively parallel RNA sequencing (RNA-seq) data were utilized in two ways. First, we obtained gene models by mapping RNA reads to the scallop genome in Tophat[17] (version 2.0.8) and by assembling the transcripts by means of Cufflinks[18] (version 2.1.1). Second, we obtained predicted gene models as other predictions by PASA using unigenes assembled by Trinity[7].

The non-redundant consensus set of gene structures was integrated in EVidenceModeler (EVM)[19] using all the gene evidence predicted above. Next, the gene models generated by EVM were filtered according to the following criteria: coding region length less than 150 bp, supported only by an *ab initio* method, and with expression value < 5 for single-exon genes and expression value < 1 for multi-exon genes. After gene filtering, 28,602 protein-coding genes were retained and constituted the final gene set for *C. farreri*.

Functional annotation of protein-coding genes was performed by means of

BLASTP (E-value threshold: 1e-05) in two integrated protein databases: SwissProt and TrEMBL. The annotation information of the best BLAST hits was retained for the scallop gene set. Protein domains were annotated by searching InterPro (v29.0) databases. To improve computing speed, we limited searches to five databases—Pfam, PRINTS, PROSITE, ProDom and SMART—using software InterProScan (version 4.7). Gene Ontology (GO) terms for each gene were retrieved from the corresponding InterPro entry. The scallop gene set was also mapped to the KEGG pathway database (release 53) to identify the best match for each gene.

Transcriptome sequencing and profiling of adult tissues/organs

Thirteen adult tissues/organs of *C. farreri* were chosen for transcriptome sequencing, including striated muscle, smooth muscle, foot, hepatopancreas, kidney, female gonad, male gonad, gill, eyes, mantle, cerebral ganglion (PGCG), and visceral ganglion (PVG). For muscle, we sampled the center region of each muscle type (striated or smooth) for transcriptome sequencing to avoid cross-tissue contamination. For each sample, transcriptome sequencing was independently conducted for three individuals (i.e., biological replicates) to ensure reliable quantification of gene expression. Total mRNA was extracted from each tissue/organ following the protocol described by Hu et al.[20]. All RNA-seq libraries were constructed using the NEB Next mRNA Library Prep Kit following the manufacturer's instructions and then were subjected to paired-end 125-bp (PE125) sequencing on the Illumina HiSeq 2000 platform.

Sequencing reads were aligned to the *C. farreri* genome using STAR aligner[21] with default parameters. Gene expression levels in terms of TPM were estimated by HTseq[22] and custom Perl scripts. Differentially expressed gene (DEG) analysis was carried out using edgeR[23] with three biological replicates, and genes with a 'fold change value ≥ 2 and adjusted p-value < 0.05 were defined as significant DEGs.

Polymorphism analysis

To characterize polymorphism in the *C. farreri* genome, reads from the sequenced individual (both 180-bp and 300-bp WGS libraries) and from five additional

resequenced individuals were aligned to the assembled genome in the BWA[6] software with the settings "-n 15 -o 1 -e 10". After that, SAMtools[24] was used to sort alignments and filter PCR duplicates. SNPs were called using SAMtools mpileup and bcftools with a minimal mapping quality of 50, and the sites with read depth lower than 4 or higher than four times the sequencing depth were filtered out. After calling, annotation of SNPs was conducted using ANNOVAR[25].

SNP density was defined as the number of SNP sites per unit region among all 6 sequenced individuals. SNP density across the genome was calculated in 1 Mb sliding windows with a step size of 50 kb, and SNP density in CDS region of each gene was also estimated. Genomic regions or CDSs with high SNP density subjected to one-sided Fisher's exact test by comparing to the corresponding chromosomal background, and the cutoff of the *p*-value was set to 1e-5. The distribution of SNP density among chromosomes or genes was visualized using the Circos software[26].

Gene family analysis and phylogenetic tree construction

The OrthoMCL pipeline[27] was used to define gene families for 13 selected species, including seven lophotrochozoans (*C. teleta*, *H. robusta*, *O. bimaculoides, L. gigantea*, *C. farreri*, *P. fucata*, and *C. gigas*), three ecdysozoans (*Strigamia maritima*, *Drosophila melanogaster,* and *Tribolium castaneum*), two deuterostomes (*B. floridae* and *Homo sapiens*), and one representative of Cnidaria (*Nematostella vectensis*) as the out-group species. The protein-coding sequences of *P. fucata*[4] were downloaded from the Marine Genomics Genome Browser (http://marinegenomics.oist.jp/pinctada_fucata/), and the datasets of other species were obtained from the Ensembl database (release 85). The longest isoform of each gene was chosen, and the genes encoding a polypeptide longer more than 30 amio acids were retained. In total, 32,843 gene families were identified via all-against-all BLASTP comparisons and clustering based on the MCL algorithm.

For phylogenetic analysis, we selected orthologous genes using a tree-based approach PhyloTreePruner[28], which screens single-gene trees and corresponding alignments to exclude paralogous sequences with evidence. After filtering short

sequences (≤100 amio acids) and potential paralogs, we obtained 1,310 orthologous groups, which were concatenated into one sequence for each species and formed a data matrix with 483,895 amino acid positions. The phylogenetic tree was constructed using RAxML[29] with the LG+Γ4 amino acid substitution model (determined by ProtTest[30]). To estimate the divergence time for *C. farreri* and other metazoans, the 1st and 2nd codon positions of the 1,310 orthologs were extracted for Bayesian dating using the MCMCtree program implemented in PAML[31]. The correlated rate model and GTR nucleotide substitution model (determined by MODELTEST[32] were selected, and the gamma rates at sites were calculated in the RAxML software (clock = 3; model = 7; alpha = 0.671). Reference divergence time values (518–581 MYA for *Homo* and *Branchiostoma*; 419–543 MYA for *Strigamia* and *Drosophila*, 450–513 MYA for *Capitella* and *Helobdella*, 307–414 MYA for *Tribolum* and *Lottia*, 531–581 MYA for *Nematostella* and *Homo*, and 500–550 MYA for *Lottia* and *Crassostrea*) retrieved from the TimeTree[33] database were used to calibrate divergence dates of other nodes on the phylogenetic tree.

The evolutionary dynamics of gene families were analyzed in software CAFÉ[34], which can identify gene families that have expanded or contracted using a stochastic birth and death model. It can estimate global parameter λ (based on the phylogenetic tree and the datasets of gene family clustering), which represents the birth and death rate of all gene families and identifies the significantly changed families (p-value < 0.05, calculated by the Viterbi method in CAFÉ). GO enrichment analysis was performed using the EnrichPipeline[35], and GO terms with false discovery rate [FDR] < 0.05 were recognized as significantly enriched.

Expressional and network analysis of muscle genes

We compared transcript abundance of various enzymes involved in glycolysis, TCA cycle and oxidative phosphorylation pathways between different types of scallop muscles or between scallop (*C. farreri*) and oyster (*C. gigas*) muscles. The sum of TPM values of genes encoding the same enzyme was defined as the expression level of a given enzyme. Differences between two types (striated and smooth) of muscles

were colored using KEGG mapper.

Coexpression gene networks were constructed by means of WGCNA[36] using 35 transcriptomes from adult tissues/organs, with the following parameters: minimum module size = 200, cutting height = 0.99, and deepSplit =F. Cytoscape[37] was used to visualize the networks. Muscle-overrepresented gene sets were identified by determining the intersection of up-regulated DEGs between muscle and all nonmuscle samples. Statistical analysis of module enrichment of muscle-overrepresented genes was conducted by the hypergeometric test ($P < 0.05$). Gene ranking by importance for the adductor muscle-related module (M3) was calculated from the strength of connection with other genes in the module and determined by intra-modular connectivity (K_{within}).

The expression profile of vertebrate muscle marker genes[38] (striated: *Mhc* (striated myosin heavy chain), *Tnnt*, *Tnni*, *Ttn* and *Zasp*; smooth: *Cnn*) in *C. farreri* was determined in three biological replicates (individuals), using the average TPM value to display the results. The average TPM value of vertebrate muscle marker genes in human was obtained from 3–5 biological replicates in the HPA dataset (http://www.proteinatlas.org/).

Characterization and evolutionary analysis of opsin genes

Putative opsin genes in *C. farreri* and other bivalves were identified by homology-based searching against the known opsin genes of other animal species retrieved from the NCBI protein database at an e-value threshold of 1e-5. For candidate genes, only those containing seven transmembrane domains and the lysine site (296K) were kept for subsequent analysis. The opsin phylogeny was constructed by the Bayesian method in software MrBayes[39] using the sequences of seven transmembrane domains, and the types of scallop opsins were determined by their phylogenetic relationships with known opsins. Ka/Ks values were estimated by means of Ka_Ks_calculator 2.0[40] using the YN method. The opsin genes of the scallop *P. yessoensis* were retrieved from the *P. yessoensis* genome database[41]. Key genes involved in rhabdomeric and ciliary phototransduction pathways were identified by

homology-based search against the known genes from *Homo* and *Drosophila* (downloaded from the NCBI protein database) at an e-value threshold of 1e-10. Putative light sensitivity of bivalve opsin genes was determined by means of amino acid combinations at key amio acid positions (164, 261, and 269) that correspond to sensitivity to different light wave lengths in human opsins. The position of the key sites was referenced to that in rhodopsin of *B. taurus*[42].

Byssal proteins and secretion regulation

For mass spectrometric analysis, proteins were extracted from byssal adhesive plaques by using the method of Miao et al.[43], and the whole protein sample as well as major SDS-PAGE fractions (Supplementary Fig. 17b) was treated with trypsin and analyzed using an Easy-nLC nanoflow HPLC system connected to an Orbitrap Elite mass spectrometer (Thermo Fisher Scientific, USA). The mass spectrometry raw data were searched against the full set of predicted proteins from the *C. farreri* genome using Mascot v2.3.0 (Matrix Science, London, UK). Searches were performed using the following parameters: trypsin as enzyme, 1 max mis-cleavage, carbamidomethyl (cysteine) as fixed modification, and oxidation (methionine) as variable modification. Protein identifications were accepted at a false discovery rate (FDR) threshold of 0.01. To be stringent, the identified proteins with ≤ 1 unique matching peptide in both datasets and with expression $ratio_{[foot/ave\_nonfoot\_organ]} \leq 2$ were excluded from further analysis. More detailed byssal protein extraction protocol and mass spectrometric analytical procedure can refer to previous studies of Miao et al.[43] and Lyu et al.[44], respectively. Functional annotation of scallop candidate BRPs was performed by means of BLASTP (E-value threshold: 1e-05) against the SwissProt protein database, and the annotation information of the best BLAST hits was retained. Domain or signal peptide annotations were performed by searching the online databases of Pfam (http://pfam.xfam.org/), InterPro (http://www.ebi.ac.uk/interpro/), SMART (http://smart.embl-heidelberg.de) and SignalP (http://www.cbs.dtu.dk/services/SignalP) under default parameters. Microstructures of the byssal thread were examined by scanning electron microscopy (Hitachi S-3400N). Among the identified BRPs, those

with foot TPM > 200 and foot_TPM/average_of_nonfoot_tissue/organ_TPM > 100 were defined as foot differentially expressed BRPs (Diff. BRPs), which were used for the subsequent comparisons of gene expression at the juvenile stage and in adult feet between *C. farreri* and *P. yessoensis*.

Forty-five RNA-seq libraries covering three foot regions (proximal, middle, and distal) and five time points after the removal of byssal threads (0, 1, 1.5, 12, and 24h) were constructed using the NEB Next mRNA Library Prep Kit following the manufacturer's instructions and subjected to PE125 sequencing on the Illumina HiSeq 2000 platform. RNA-seq reads were mapped to the *C. farreri* genome in Tophat[17] (ver 2.0.9). Overrepresented genes in each foot region (proximal, middle, or distal) were identified by DEG analysis using the edgeR package[23], i.e., by comparing gene expression between one region and the other two regions.

A nitro blue tetrazolium (NBT) staining assay was performed on the whole byssal threads. After attachment to frosted glass for 24 h, byssal threads were harvested and incubated with NBT glycinate for ~10 min before visualization. A catechol oxidase assay for *in situ* detection of tyrosinase activity was carried out by incubation of foot slices overnight with 20 mM L-tyrosine in PBS followed by examination under a Nikon SMZ25 stereomicroscope with a 2× objective lens. For phylogenetic analysis of tyrosinases, a maximum likelihood (ML) tree was constructed in RAxML[29] with the best fitting model of WAG+I+G+F determined by ProtTest[30] for amino acid substitution. All the positions containing gaps and missing data were eliminated and the robustness of the tree was tested by reanalysis of 1,000 bootstrap replicates.

Neurotoxin resistance analysis and TR network construction

The voltage-gated sodium channel protein (*Nav*) sequences of four selected animals (*H. sapiens, Tetraodon nigroviridis, Takifugu rubripes*, and *D. melanogaster*) were downloaded from the NCBI protein database, and the homologous proteins in the scallop *C. farreri* and other bivalves were identified via searches involving these known *Nav* sequences against bivalve genomes using the BLAST algorithm with an e-value threshold of 1-e10. Amino acids positions putatively conferring PST and TTX

resistance were identified based on conservation of previously reported sites[45-50]. Expression levels of *Nav1* and *Nav2* in *C. farreri* in major tissues or organs were determined on the basis of RNA-seq data, normalized, and presented in the form of TPM.

To analyze effects of PST-producing dinoflagellates on scallop gene expression, 2-year-old adult scallops of *C. farreri* were acclimated and depurated for three weeks and then fed a toxic strain of the marine dinoflagellate *A. minutum*. Each scallop was fed once a day with *A. minutum* concentration of 2500 cells/mL in a 3-L volume. Scallops were sampled on day 0 (control), and after 1, 3, 5, 10, and 15 days of toxic-alga exposure, three individuals at each time point, and PST concentrations in tissues of the sampled scallops were determined by a high-performance liquid chromatography with tandem mass spectrometry (HPLC-MS/MS) method[51].

Thirty-six RNA-seq libraries of the hepatopancreas and kidney from *C. farreri* fed with *A. minutum* were constructed using the NEB Next mRNA Library Prep Kit following the manufacturer's instructions and subjected to PE125 sequencing on the Illumina HiSeq 2000 platform. RNA-seq reads were mapped to the *C. farreri* genome using Tophat (ver 2.0.9)[17], and the expression levels of all the genes were normalized and presented in the form of TPM. DEGs were identified using R package edgeR[23] ($P < 0.05$, ANOVA). The coexpression gene networks for the hepatopancreas and kidney were constructed using the R package WGCNA[36], with the following parameters: minimum module size = 200, cutting height = 0.99, and deepSplit =F. Cytoscape[37] was employed for visualization of the coexpression networks. Over-representation analysis of the genes that were differentially expressed between toxin-exposed and control groups was performed for each module by a hypergeometric test ($p < 0.05$) to identify TR modules. Heat maps of the expression patterns of TR modules for the kidney and hepatopancreas were drawn using custom R scripts. GO enrichment analysis of each module in the networks was conducted using the EnrichPipeline[35].

The cytosolic sulfotransferase (*Sult*) genes were identified in the genomes of three bivalves (*C. farreri*, *C. gigas*, and *P. fucata*), *H. sapiens* and *D. melanogaster* using BLAST in public databases (SwissProt and TrEMBL) with an e-value of 1e-5

and were confirmed by comparing with the Conserved Domains Database. The ML tree of SULTs was constructed in RAxML[29] with the best-fitting model of G+LG determined by ProtTest[30] for amino acid substitution. All positions containing gaps and missing data were eliminated and the robustness of the tree was tested by reanalysis of 1,000 bootstrap replicates.

Supplementary Figures

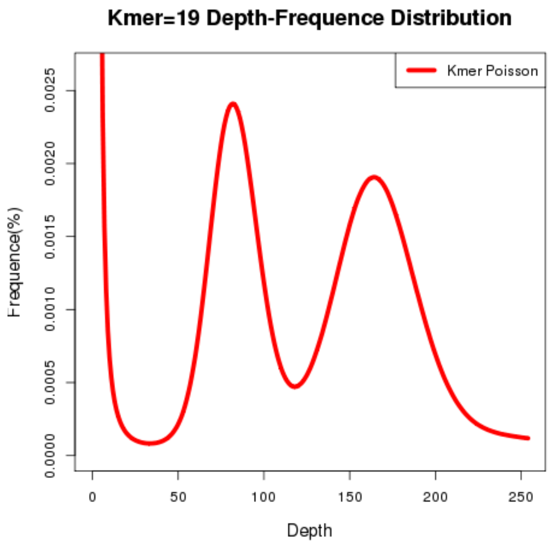

Supplementary Figure 1 | Distribution of 19-mer frequency in the *C. farreri* genome.

High-quality sequencing reads generated from 180-bp WGS libraries were used to generate the 19-mer depth distribution curve. Two peaks rather than one were observed, indicating high genomic heterozygosity.

1) gi|348020200|gb|JN703459.1| *Chlamys farreri* clone BAC Bam040H03, complete sequence

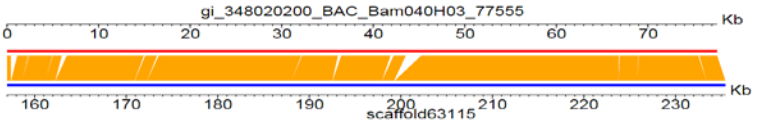

2) gi|348020199|gb|JN703458.1| *Chlamys farreri* clone BAC Bam123C08, complete sequence

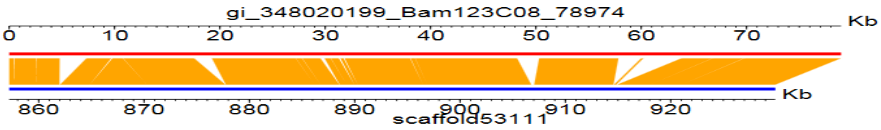

3）gi|348020201|gb|JN703460.1| *Chlamys farreri* clone BAC Bam187A08, complete sequence

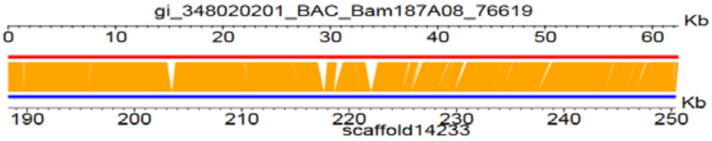

4) BB240C9 (chpaxa)

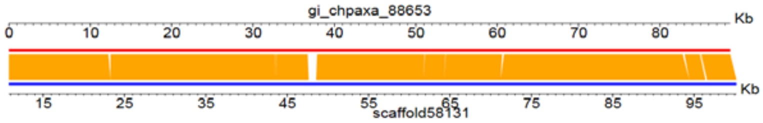

Supplementary Figure 2 | Sequence alignments among three BAC clones and the *C. farreri* genome assembly, showing 99-100% BAC coverage by the assembly.

Orange blocks denote positively aligned regions, with blanks representing unmapped gaps. BACs named "Bam*" were derived from another study[52], and the last one was sequenced in this study.

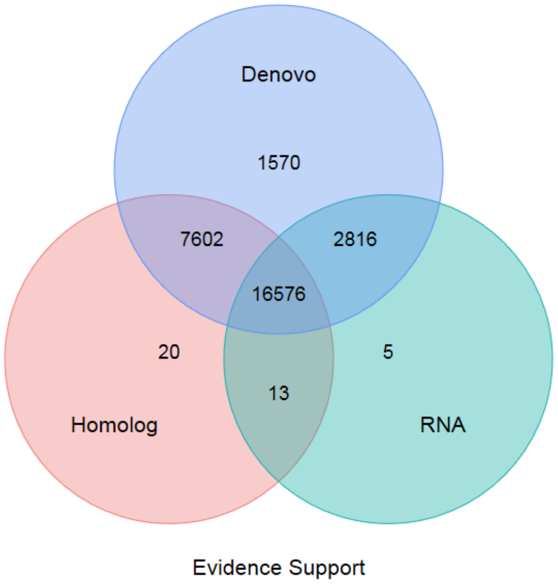

Supplementary Figure 3 | Comparison of gene sets obtained by different prediction methods (*de novo*, homology-based, and RNA-based).

A total of 28,602 genes were predicted, over 94% of which are supported by at least two prediction methods.

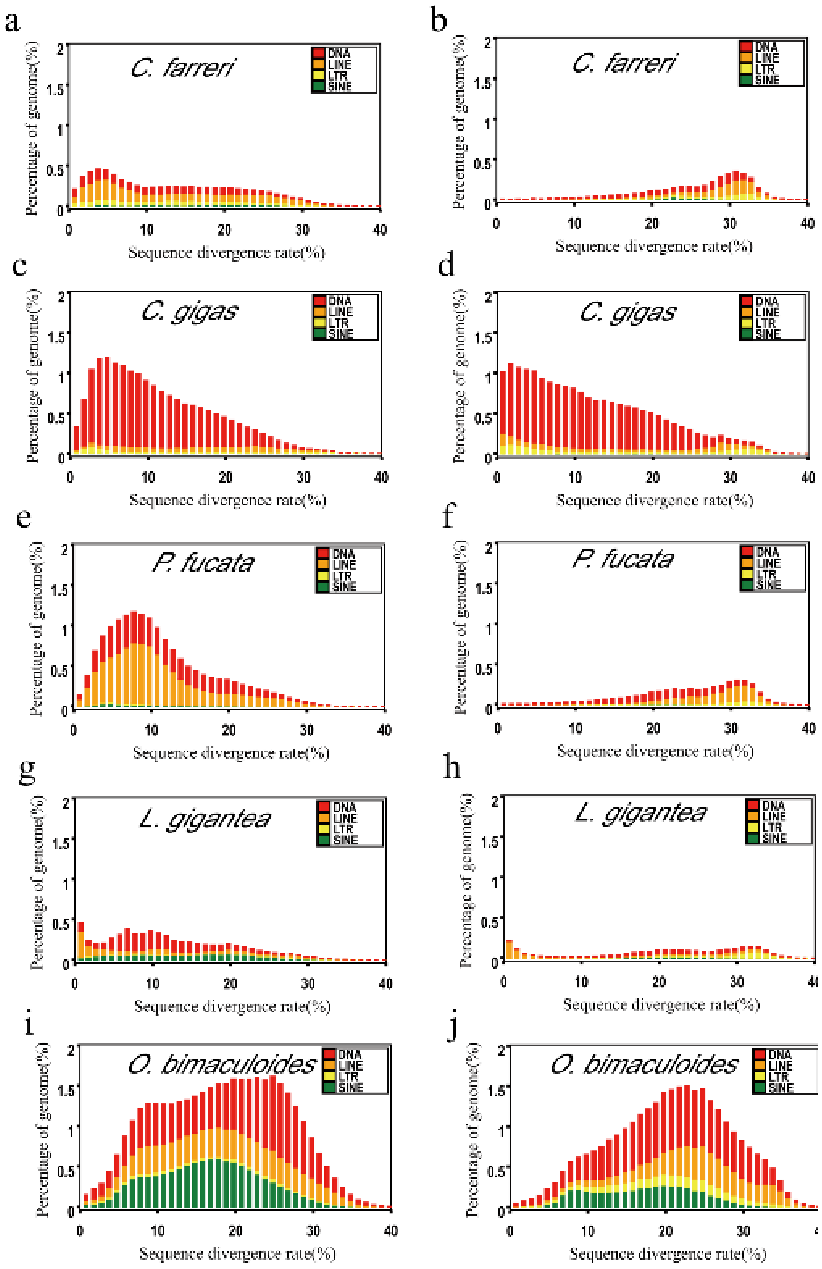

Supplementary Figure 4 | Divergence rates of transposable elements (TEs) identified by homology (a, c, e, g, and i) or de novo prediction (b, d, f, h, and j) in the scallop genome and genomes of four other mollusk species.

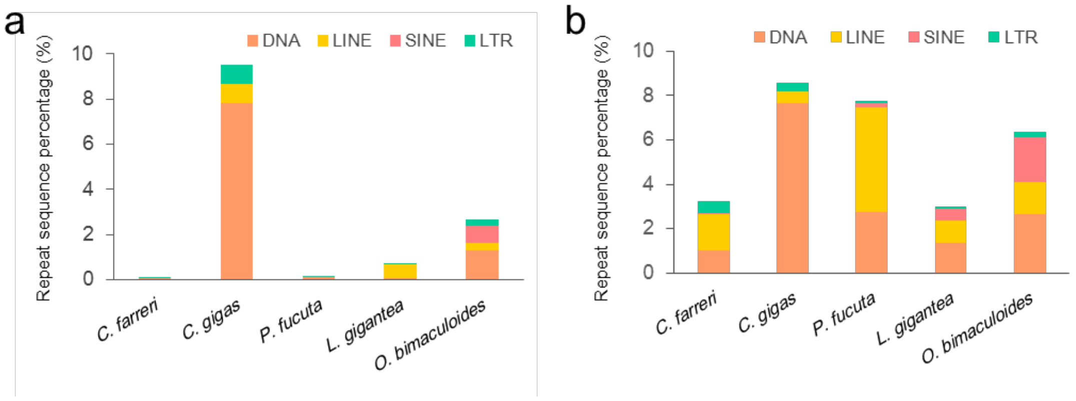

Supplementary Figure 5 | Distribution of TEs with a divergence rate < 10%.

The scallop *C. farreri* shows a lower proportion of young TEs with a divergence rate < 10% than other molluscs do according to either homolog-based (a) or *de novo* prediction (b).

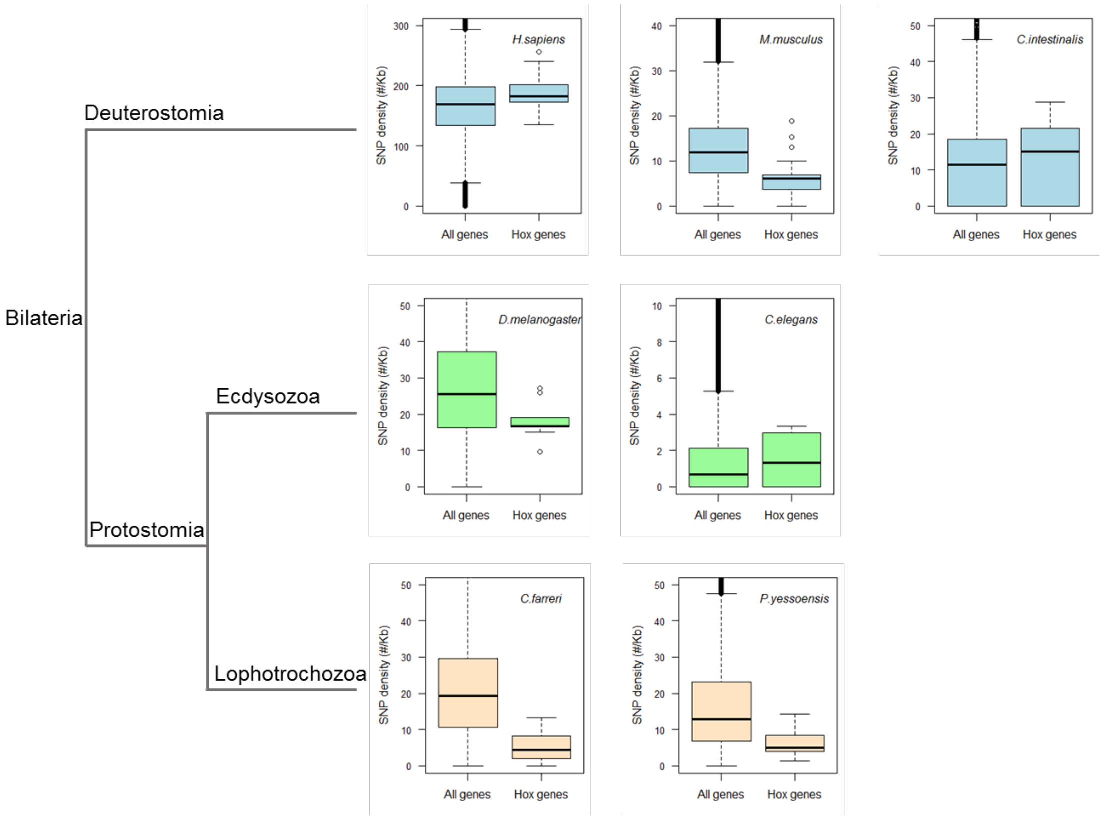

Supplementary Figure 6 | Distribution of SNP density in the CDS regions of all genes and *Hox* genes in seven selected animal species representing major phylogenetic groups.

Scallop Hox genes are largely devoid of polymorphism. Similar patterns are also observed in the fruit fly *D. melanogaster* and mouse *Mus musculus*, but not in all analyzed species. The SNP data from *P. yessoensis* were derived from our unpublished resequencing results, and those of other species were retrieved from the NCBI dbSNP database (Accession IDs: human_9606_b149_GRCh38p7, mouse_10090, squirt_7719, fruitfly_7227 and nematode_6239).

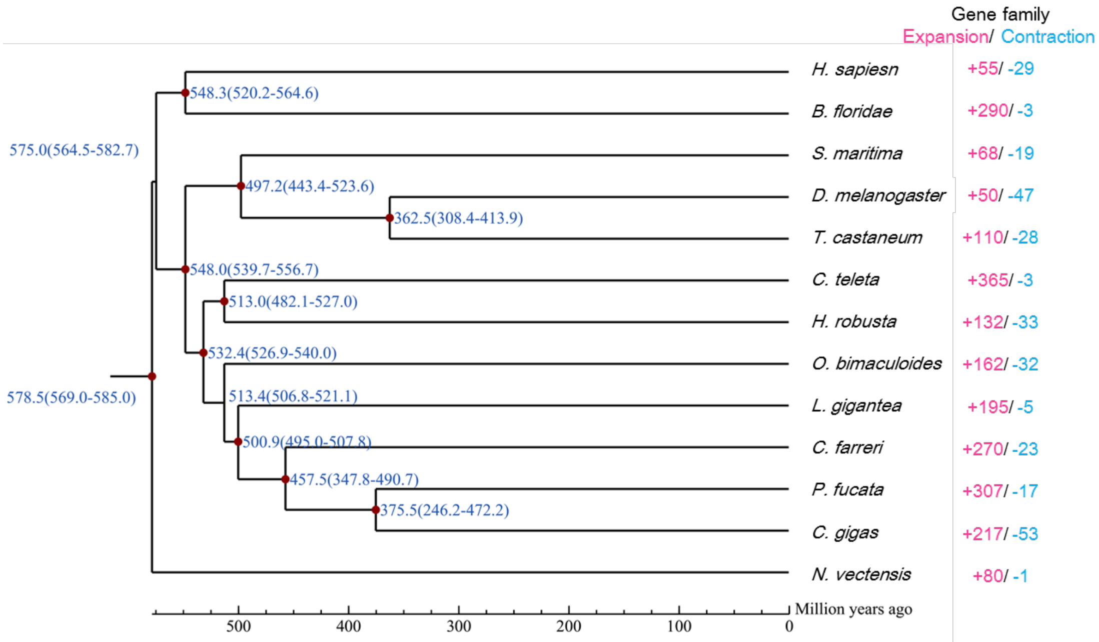

Supplementary Figure 7 | Phylogenetic tree and gene family expansion/contraction analysis for 13 selected animal species.

The phylogenetic tree was built by means of 1,310 orthologous genes. Molecular dating analysis suggests that the scallop lineage diverged from the lineage leading to *C. gigas* and *P. fucata* ~457 MYA, and Bivalvia diverged from its sister group Gastropoda ~500 MYA. Gene family analysis shows that 270 gene families are significantly expanded in the scallop relative to other bivalves. The numbers refer to gene families showing expansion or contraction.

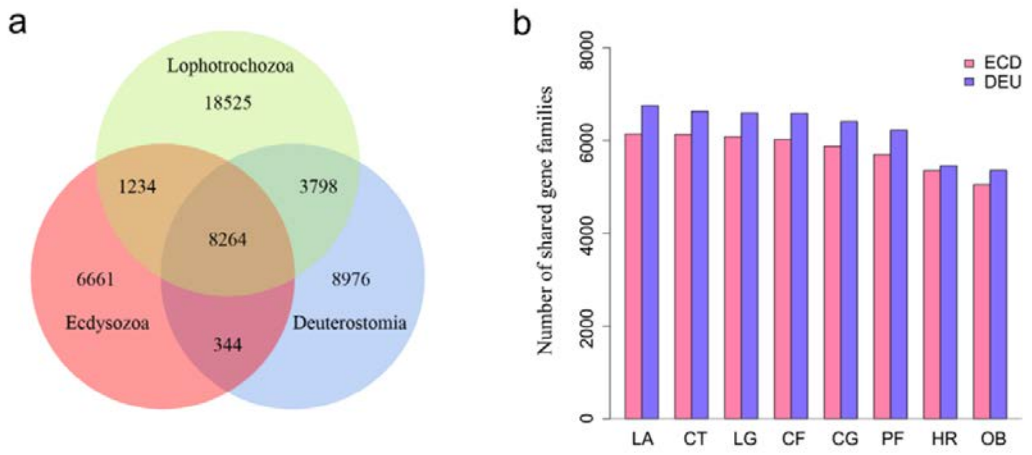

Supplementary Figure 8 | Gene family comparison among major bilaterian groups and within Lophotrochozoa.

(a) Lophotrochozoans share substantially more gene families (12,062) with deuterostomes than ecdysozoans (9,498), suggesting that lophotrochozoan genomes are less derived than those of ecdysozoans. (b) Among bivalves, scallop *C. farreri* shares the most gene families (7,604 in common) with deuterostomes and ecdysozoans; this result is comparable to that (7,788) for the brachiopod *Lingula anatina*, a "living fossil" lophotrochozoan. Species abbreviations: LA, *L. anatina*; CT, *Capitella teleta*; LG, *Lottia gigantea*; CF, *Chlamys farreri*; CG, *Crassostrea gigas*; PF, *Pinctada fucata*; HR, *Helobdella robusta*; OB, *Octopus bimaculoides.*

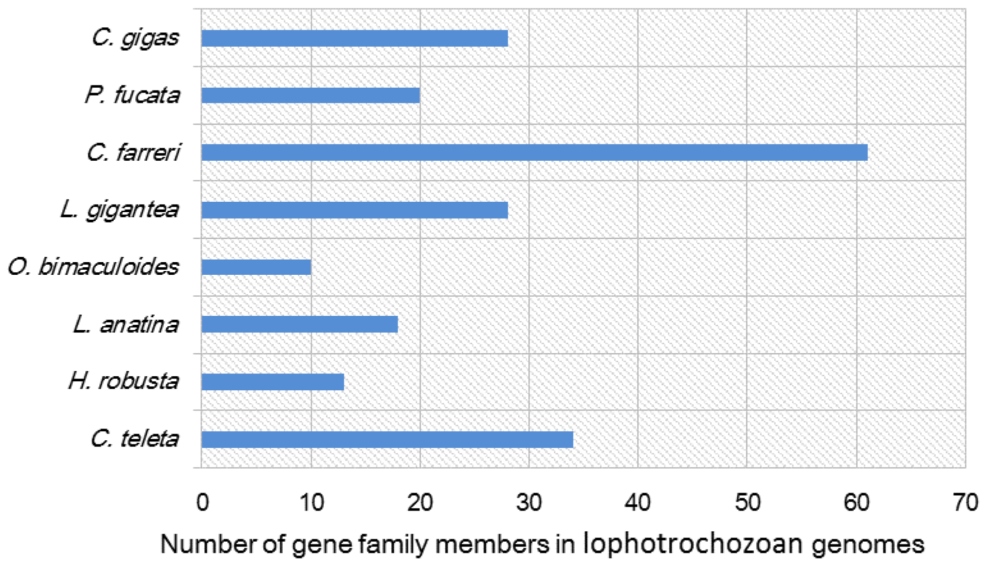

Supplementary Figure 9 | The number of sodium- and chloride-dependent neurotransmitter transporter genes in eight lophotrochozoan genomes.

Among eight lophotrochozoans, scallop *C. farreri* exhibits remarkable expansion of this gene family.

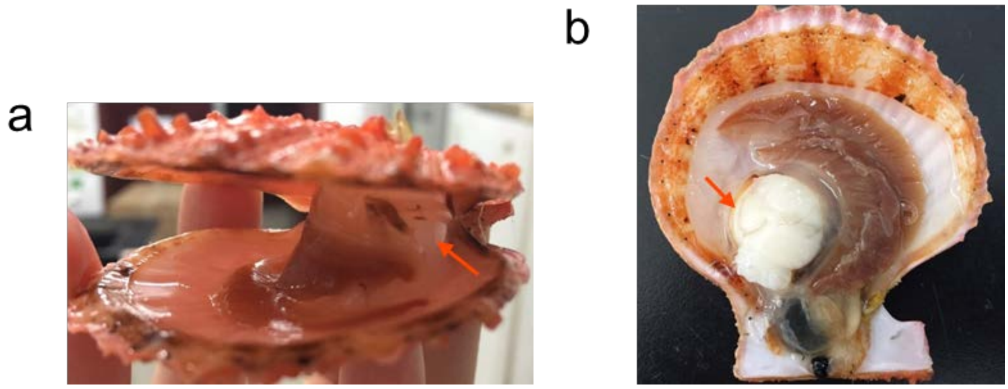

Supplementary Figure 10 | The large adductor muscle of the Zhikong scallop *C. farreri*.

The red arrows indicate the adductor muscle of a live (a) and an opened (b) scallop.

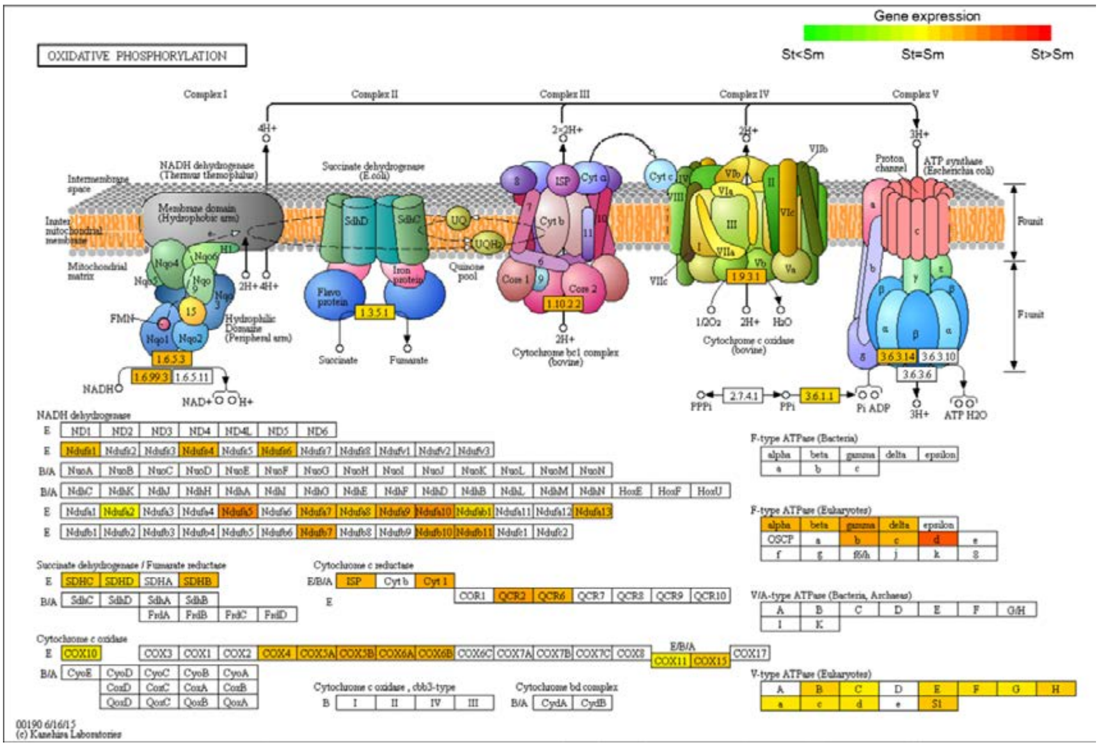

Supplementary Figure 11 | Relative expression of oxidative-phosphorylation-related genes in striated (St) and smooth (Sm) muscles of *C. farreri*.

Most genes in this pathway show higher expression in striated than smooth muscle.

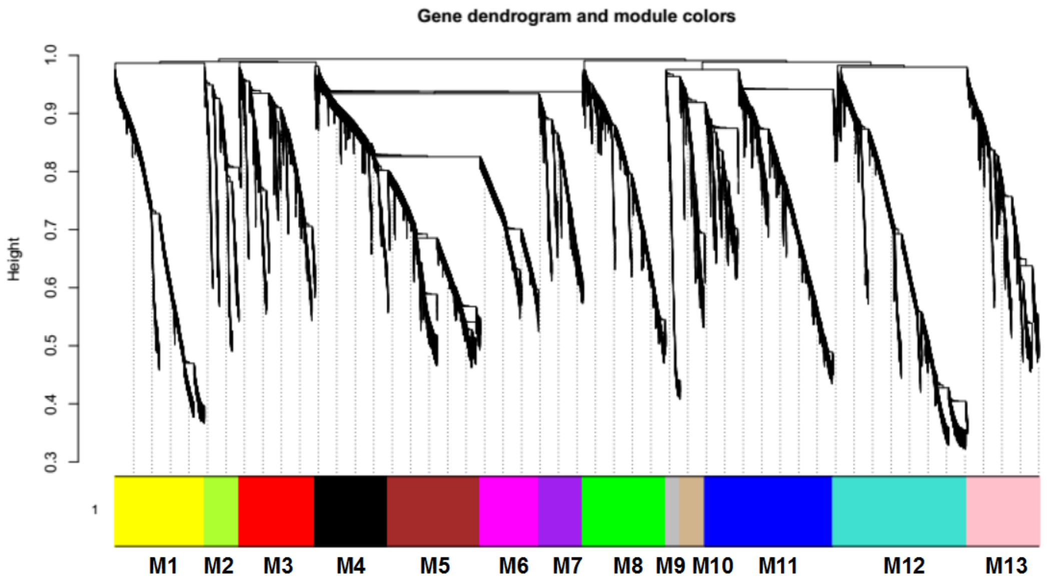

Supplementary Figure 12 | A gene coexpression network constructed using 35 transcriptomes from adult scallop organs.

Dendrograms were produced by average linkage hierarchical clustering of genes on the basis of a topological overlap. Horizontal color bars represent different modules of coexpressed genes. Unassigned genes are indicated in grey.

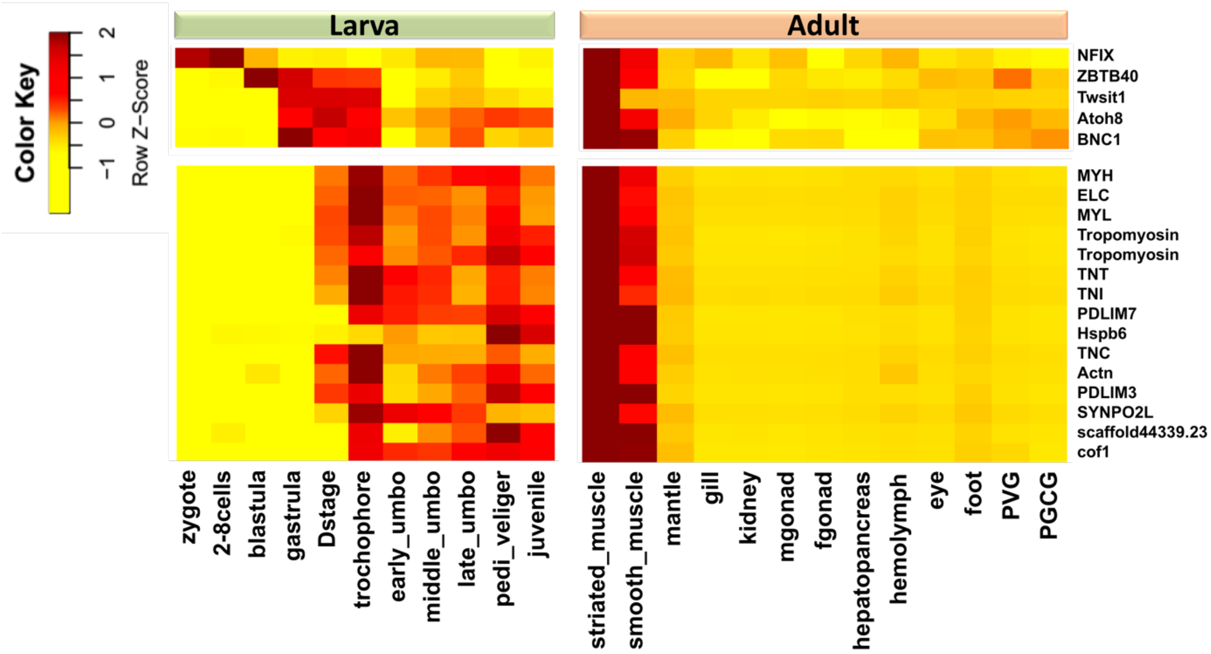

Supplementary Figure 13 | Expression profiles of top 5 transcription factor genes (*Nfix*, *Zbtb40*, *Twist1*, *Atoh8*, and *Bnc1*) and muscle differentiation genes in adductor muscle-related module M3.

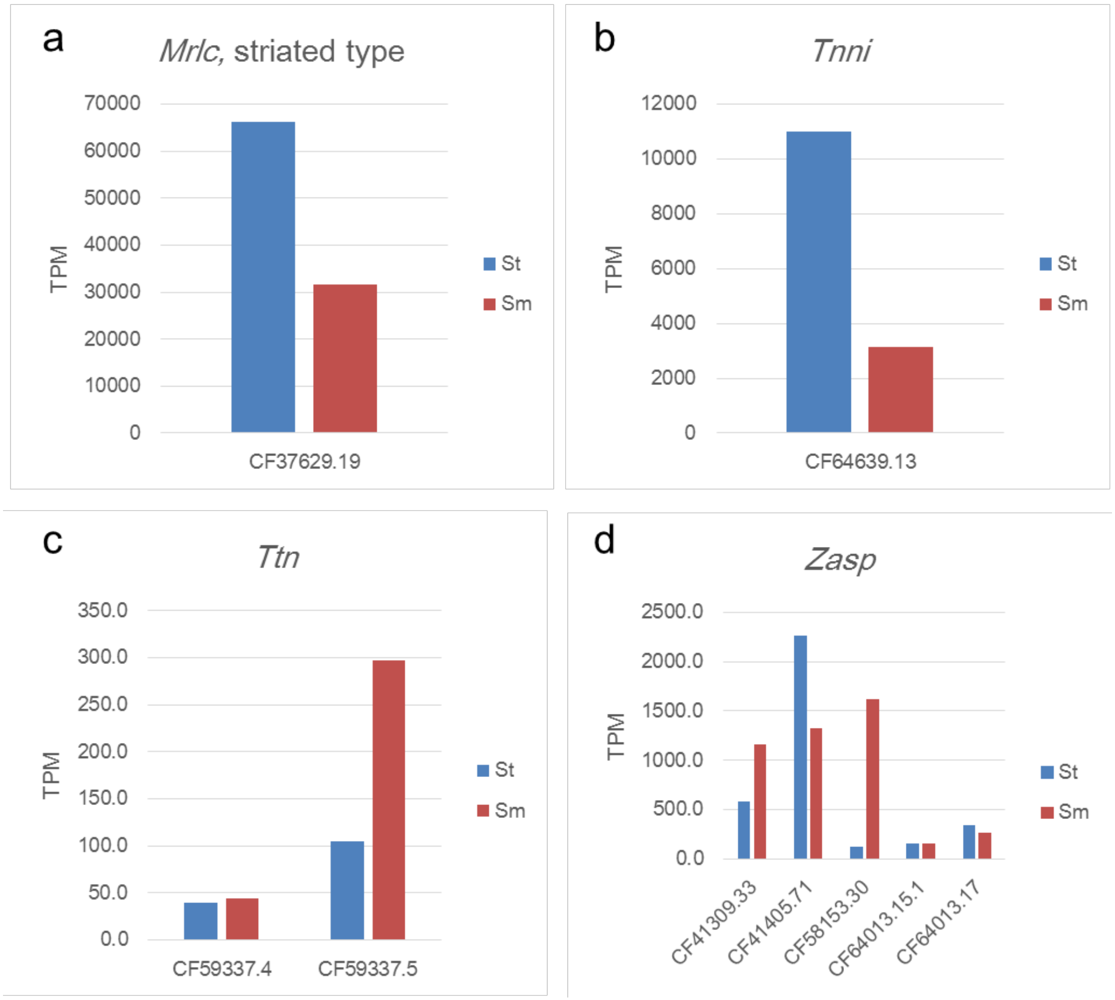

Supplementary Figure 14 | Expression profiles of vertebrate striated-muscle marker genes in striated (St) and smooth (Sm) muscles of *C. farreri*.

Vertebrate striated-muscle-specific marker genes show high expression in both striated and smooth muscles of *C. farreri*.

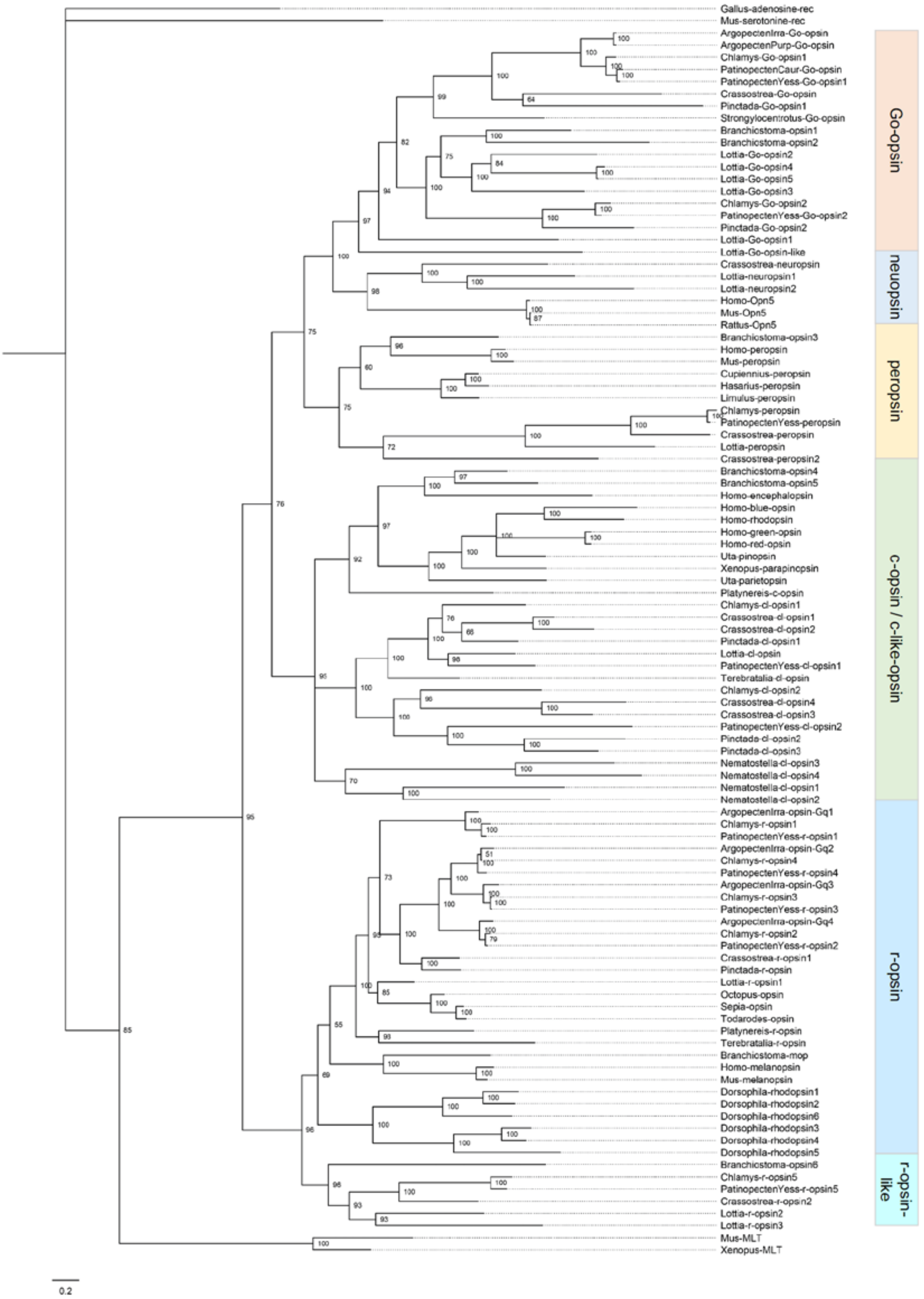

Supplementary Figure 15 | A phylogenetic tree of opsin genes.

The phylogeny of opsin genes was constructed by the Bayesian method with sequences of seven-transmembrane helices. The posterior probability is indicated at the tree nodes. Opsin genes of *Chlamys farreri*, *Patinopecten yesoensis*, *Pinctada fucata*, *Crassostrea gigas* and *Lottia gigantea* are derived from their corresponding genomes: CF: r-opsin1, CF63953.11; r-opsin2, CF65015.35; r-opsin3, CF65015.36; r-opsin4, CF65015.34; r-opsin5, CF55503.5; cl-opsin1, CF50279.10; cl-opsin2,

CF64439.21; Go-opsin1, CF16535.118; Go-opsin2, CF7665.7; peropsin, CF 8905.11. PY: r-opsin1, PY_T05889; r-opsin2, PY_T16017; r-opsin3, PY_T16018; r-opsin4, PY_T16016; r-opsin5, PY_T05307; cl-opsin1, PY_T16615; cl-opsin2, PY_T23365; Go-opsin1, PY_T06096; Go-opsin2, PY_T07058; peropsin, PY_T00595. PF: r-opsin, aug2.0_566.1_20913.t1; cl-opsin1, aug2.0_241.1_03869.t1; cl-opsin2, aug2.0_3710.1_02475.t1; cl-opsin3, aug2.0_2820.1_02262.t1; Go-opsin1, aug2.0_2001.1_05355.t1; Go_opsin2, aug2.0_1914.1_15309.t1. CG: r-opsin, CGI_10013541; cl-opsin1, CGI_10008682; cl-opsin2, CGI_10008683; c-lopsin3, CGI_10005836; cl-opsin4, CGI_10005837; Go-opsin, CGI_10023806; peropsin1, CGI_10021860; peropsin2, CGI_10021365; neuropsin, 10017784. *Lottia gigantea*: r-opsin1, 108602; r-opsin2, 154846; r-opsin3, 156440; cl-opsin, 64356; Go-opsin1, 173452; Go-opsin2, 156508; Go-opsin3, 116468; Go-opsin4, 123097; Go-opsin5, 122769; Go-opsin like, 152675; peropsin, 154374; neuropsin1, 140119; neuropsin2, 72363. Go-opsin sequences of *Argopecten purpuratus* and *Patinopecten caurinus* are derived from our unpublished data. Accession numbers of other opsin protein genes are as follows: *Homo sapiens*: blue opsin, P03999; green opsin, P04001; red opsin, P04000; rhodopsin, NP_000530; melanopsin, NP_150598; encephalopsin, NP_055137; peropsin, NP_006574; Opn5, Q6U736. *Rattus norvegicus*: Opn5, NP_861437. *Mus musculus*: melanopsin, AAF24979; peropsin, AAC53344; Opn5, NP_861418. *Drosophila melanogaster* (DM): opsin-Rh1, AAB31029.1; opsin-Rh2, AAA28734.1; opsin-Rh3, AAB31032.1; opsin-Rh4, AAA28856.1; opsin-Rh5, AAB38966.1; opsin-Rh6, NP_524368.4. *Argopecten irradians* (AI): Go-opsin1, APB88014.1; Go-opsin2, APB88015.1. *Uta stansburiana*: parietopsin, DQ100320; pinopsin, DQ100321. *Xenopus tropicalis*: parapinopsin, BAD17960. *Branchiostoma belcheri*: opsin1, BAC76019; opsin2, BAC76020; opsin3, BAC76023; opsin4, BAC76021; opsin5, BAC76022; opsin6, BAC76024; melanopsin, Q4R1I4. *Strongylocentrotus purpuratus*: Go-opsin, XP_783329. *Terebratalia transversa*: r-opsin, AGJ70280; cl-opsin, ADZ24786. *Sepia officinalis*: opsin, O16005. *Todarodes pacificus*: opsin, P31356. *Limulus polyphemus*: peropsin, AIT75833. *Hasarius adansoni*: peropsin, BAJ22674. *Cupiennius salei*: peropsin, CCP46949. Out-group sequences: *Mus musculus*: MLT, O88495; rec, NP_766400.1. *Gallus gallus*: adenosine-rec, NP_990418.1. *Xenopus laevis*: MLT, OCT65936.1. The scale bar represents 0.2 amino acid substitutions per site.

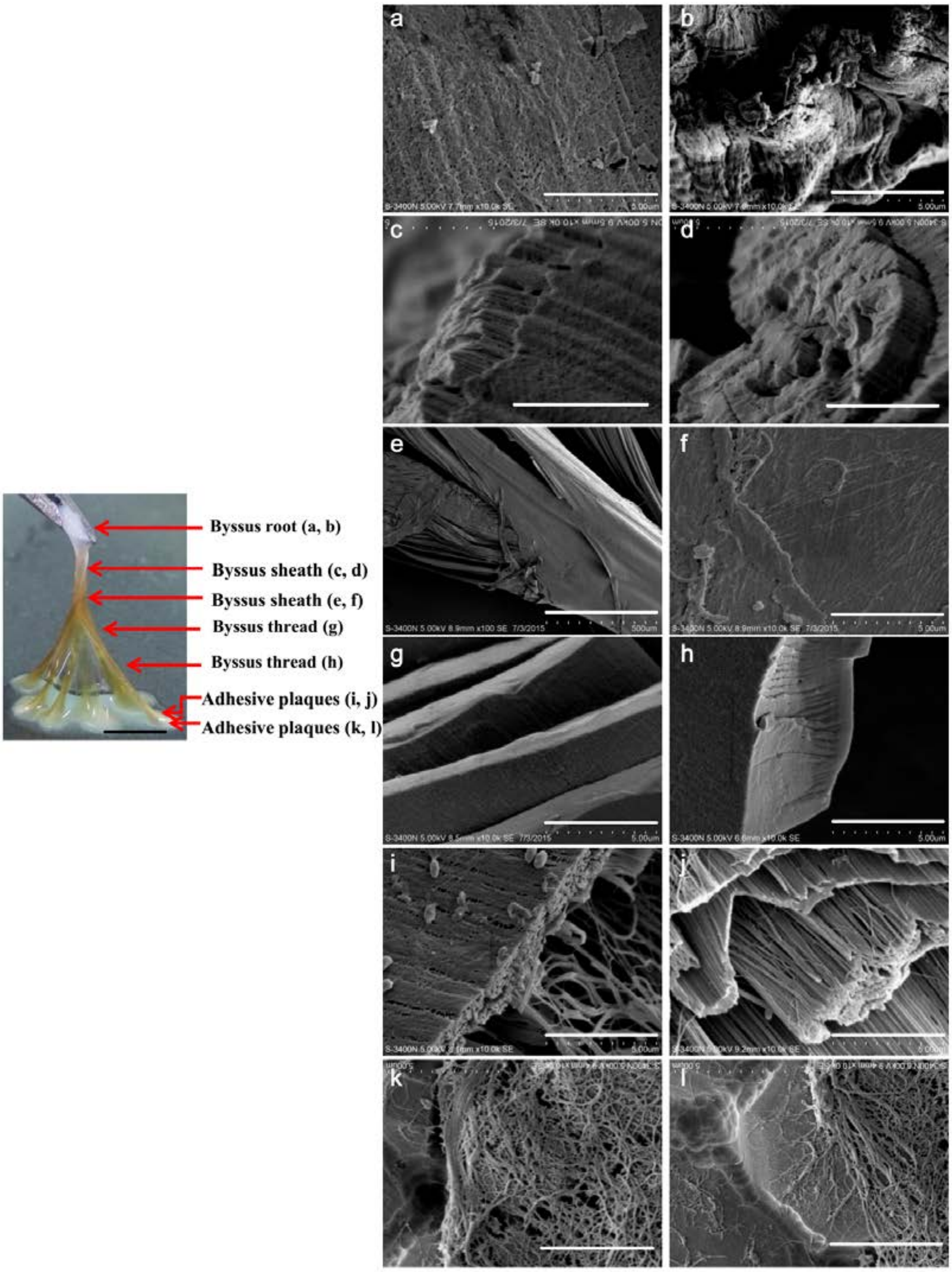

Supplementary Figure 16 | SEM photographs of *C. farreri* byssal threads.

(a) Surface structure of a byssus root showing many holes. (b) Cross-sectional structure of a byssus root. The inner part of the byssus root has loose and reticular structure, where proteins intertwine. (c,d) Cross-sectional structure of a byssus sheath, which is multilayered, and the number of sheaths equals that of the byssus thread. All sheaths are arranged in concentric circles. Highly folded proteins are distributed between two layers of a sheath, as well as in the center of the circle. (e, f) Surface

structure of a byssus sheath. The sheath is a dense protein layer, corresponding to a bunch of byssus threads. (g, h) Cross-sectional structure of byssus threads. Each bundle of byssus threads has a dense plate-like structure. (i, j) Cross-sectional structure of byssal adhesive plaques, which are composed of a filamentous and adhesive layer, not completely dispersed. (k, l) Surface structure of byssal adhesive plaques. Scale bar represents 5 mm for the left picture, while for SEM photographs, scale bars represent 500 μm for (e) and 5 μm for all the others.

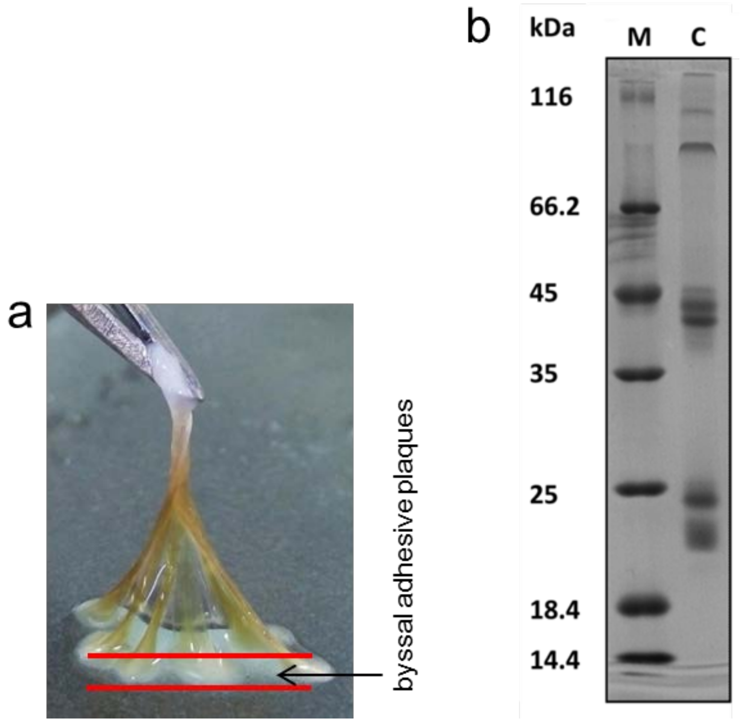

Supplementary Figure 17 | A photograph of the byssi and SDS-PAGE analysis of byssal proteins extracted from the byssal adhesive plaques of *C. farreri*.

(a) The photograph of the byssi secreted by *C. farreri*. The region between red lines indicates the byssal adhesive plaques. (b) The scallop's byssal proteins extracted from plaques (lane C). The byssal proteins are in three major fractions by molecular weight. Lane M is the protein molecular weight markers and molecular weights are indicated.

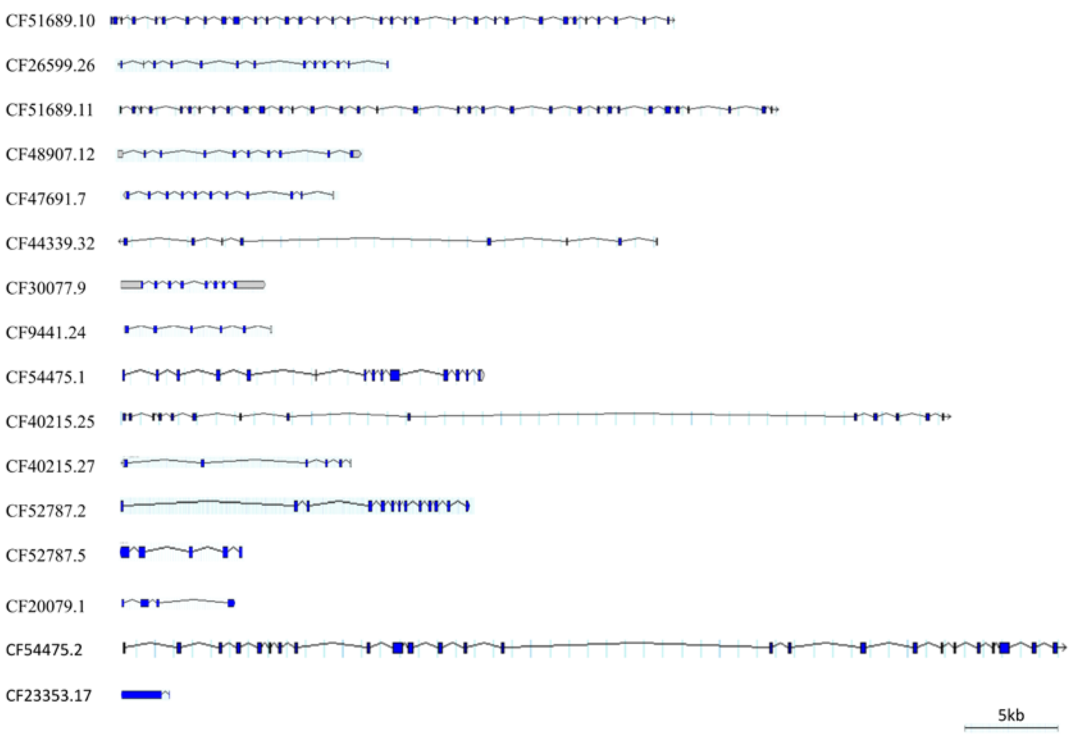

Supplementary Figure 18 | Schematic presentation of the gene structures of 16 scallop BRPs.

The gene structures were retrieved from the gbrowser page of the *C. farreri* genome website (http://mgb.ouc.edu.cn/cfbase/cgi-bin/gb2/gbrowse/cf). Exons are shown as blue boxes; the 5' and 3' UTR are shown as grey boxes; and the lines depict the introns.

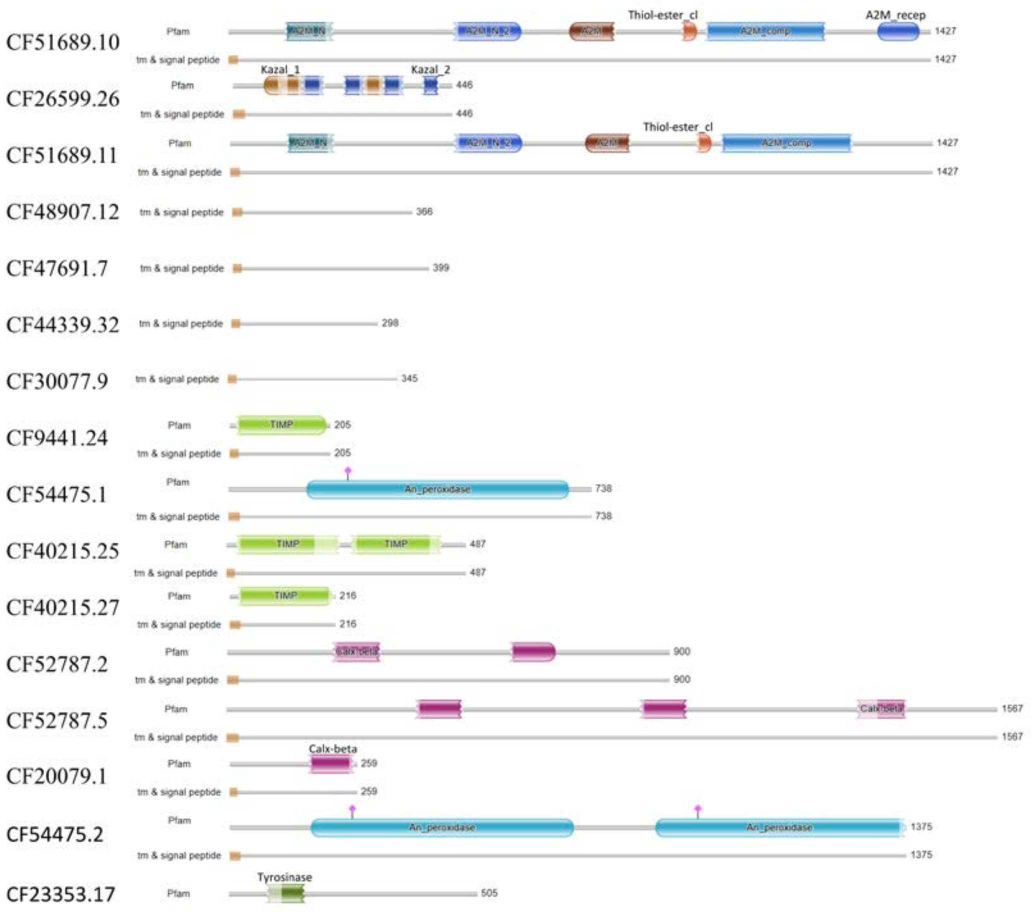

Supplementary Figure 19 | Schematic presentation of protein domains of 16 scallop BRPs.

The domain annotations from the Pfam database (http://pfam.xfam.org/) are shown here, and those from other databases (InterPro, SMART and SignalP) are available in Supplementary Data 4.

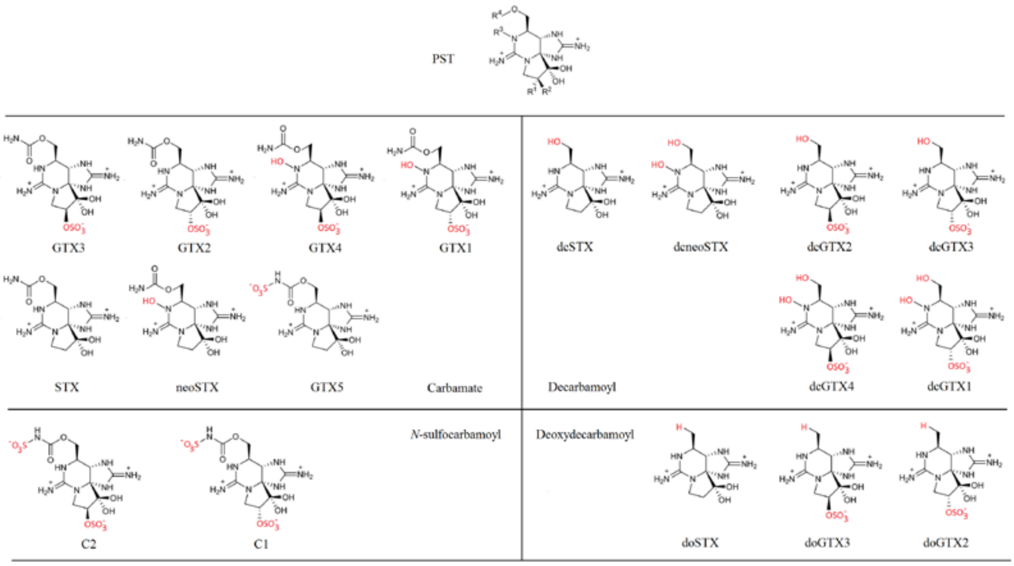

Supplementary Figure 20 | Molecular structures of paralytic shellfish toxins (PSTs).

Saxitoxin (STX) and its derivatives, including N-sulfocarbamoyl toxins (C1/2), neosaxitoxin (NeoSTX) and gonyautoxins (GTX), are collectively referred to as PSTs.

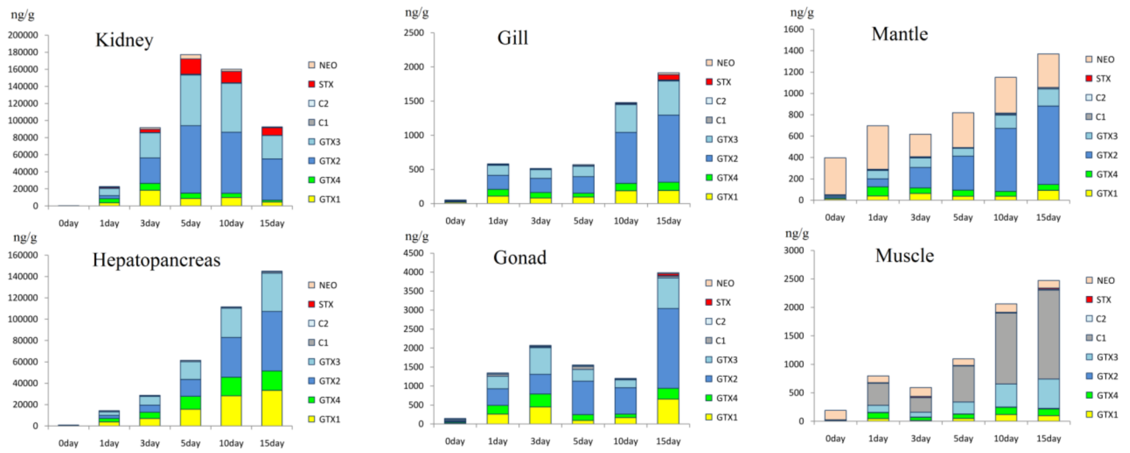

Supplementary Figure 21 | Temporal profiles of PSTs in six organs of *C. farreri* during feeding with *Alexandrium minutum*.

Eighteen individuals were sampled at six time points (0, 1, 3, 5, 10, and 15 days), three per time point, after exposure to toxic *Alexandrium minutum*. The composition and concentration of PSTs in each organ were analyzed by HPLC for each PST. The average concentration among the three replicate individuals (biological replicates) is shown.

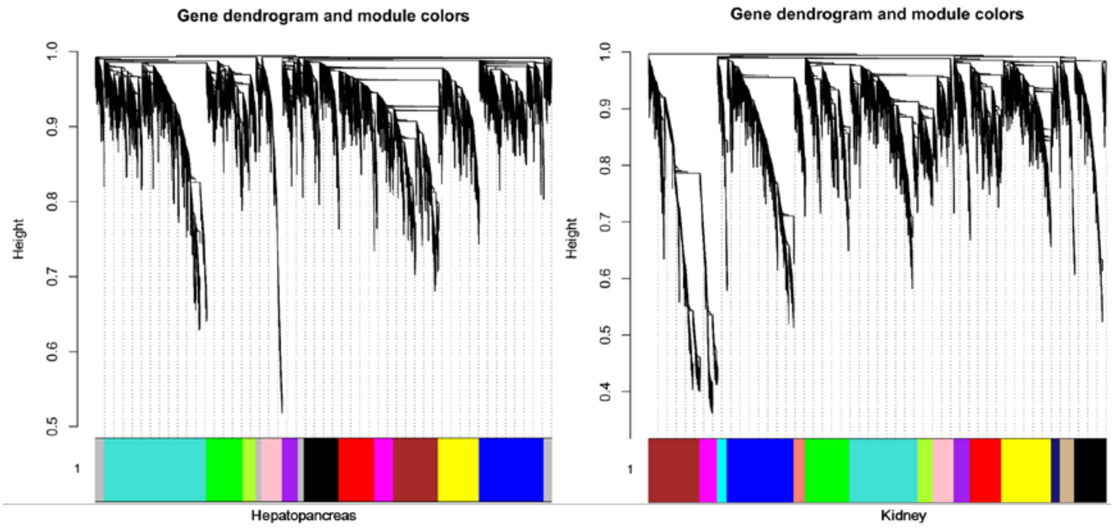

Supplementary Figure 22 | Gene regulatory networks and modules for the *C. farreri* hepatopancreas and kidney after feeding with *A. minutum*.

Dendrograms were produced by average linkage hierarchical clustering of genes on the basis of a topological overlap. Horizontal color bars represent different modules of coexpressed genes. Unassigned genes are indicated in grey.

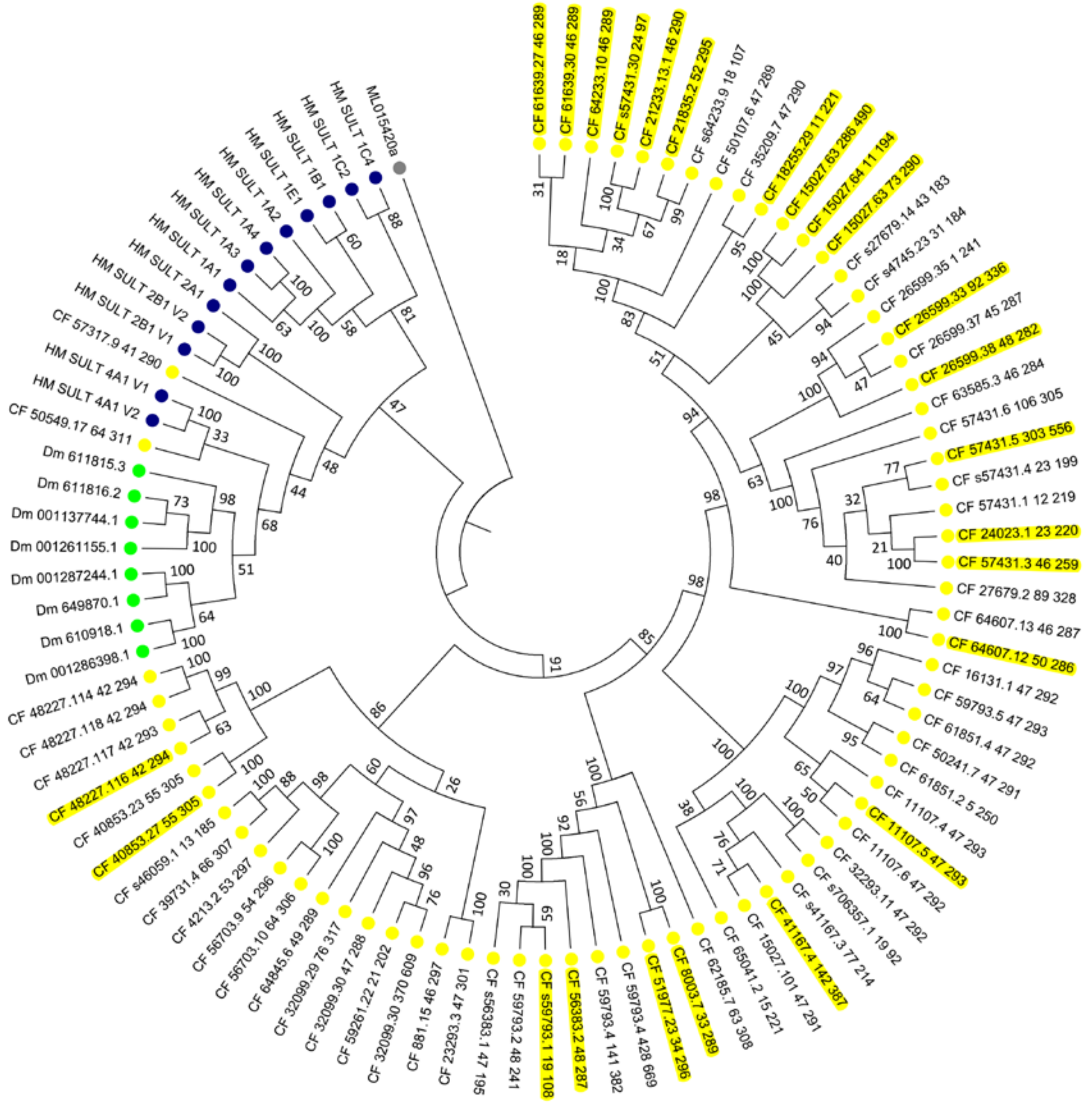

Supplementary Figure 23 | Phylogenetic analysis of *C. farreri* sulfotransferase (SULT) proteins.

The phylogeny of SULTs was constructed using conserved domains by the maximum likelihood (ML) method implemented in RAxML, according to the amino acid substitution model LG + G with 1000 runs for bootstrap support. Among the 83 SULTs in the scallop genome, only two are clustered with those of humans and the fruit fly, whereas the others are clustered in a group that is specific to the scallop, indicating expansion of the *Sult* gene family in the scallop. Numbers above the branches are support percentages for 1,000 bootstrap replicates. Genes with a yellow background are those showing significantly differential expression after exposure to *A. minutum* ($P < 0.05$, ANOVA), all belonging to the expanded group. Abbreviations: CF, *C. farreri*; HM, *H. sapiens*; Dm, *D. melanogaster*; ML, *Mnemiopsis leidyi*. The *Sult* gene from the ctenophore serves as the outgroup.

Supplementary Tables

Supplementary Table 1 | Statistics of the genome sequencing data of *C. farreri*.

Pair-end libraries	Insert size	Clean data (Gb)	Read length (bp)	Sequencing depth (×)[a]
Illumina data	180 bp	143.14	100/100	150.68
	300 bp	26.78	100/100	28.19
	500 bp	72.28	100/100	76.08
	2 Kbp	29.06	100/100	30.59
	5 Kbp	32.63	100/100	34.35
	10 Kbp	28.29	100/100	29.78
	20 Kbp	12.94	100/100	13.62
	30 Kbp	17.66	100/100	18.59
Total	-	362.78	-	381.88

[a] Sequencing depth was calculated based on the genome size of 950 Mb according to k-mer analysis.

Supplementary Table 2 | Summary statistics of the assembled *C. farreri* genome.

Estimated genome size	950 Mb
Number of chromosomes	19
Sequencing depth	381.88×
Assembled genome size	779.9 Mb
Contig N50	21.5 Kb
Scaffold N50	602 Kb
Percentage of scaffolds anchored to chromosome	63.86%
Mapped reads (%)	95.82
ESTs covered by assembly (%)	99.6~100
GC content (%)	35.49
Repeat rate (%)	32.07
Number of protein-coding genes	28,602
Mean gene length	11,130 bp
Mean exon length	215 bp
Mean intron length	1,734 bp
Average exon number per gene	7

Supplementary Table 3 | Statistics of the *C. farreri* genome assembly.

	Contig		Scaffold	
	Size (bp)	Number	Size (bp)	Number
N90	3,102	41,247	18,710	2,589
N80	7,691	26,639	141,875	1,098
N70	11,961	18,938	284,253	717
N60	16,449	13,647	434,990	498
N50	21,500	9,690	602,055	344
Total	745,399,745	148,999	779,935,877	96,024

Only scaffolds >= 200 bp are included in the genome assembly

Supplementary Table 4 | Alignment of four BAC clones to the assembled *C. farreri* genome.

BAC type	BAC ID	Scaffold ID	BAC Length (bp)	BAC coverage by scaffold (%)[a]
CF	Bam040H03	Scaffold63115	77,555	100
CF	Bam123C08	Scaffold53111	78,974	100
CF	Bam187A08	Scaffold14233, Scaffold54313	76,619	99.89*
CF	BB240C9	Scaffold58131	88,653	100

BACs named "Bam*" are derived from a previous study[1], and the last one is sequenced by this study.

[a]If a BAC is covered by multiple scaffolds, the cumulative coverage percentage from all scaffolds with over 10% BAC coverage is shown.

Supplementary Table 5 | Integrity evaluation of the *C. farreri* assembly by read remapping analysis.

Reads	
Mapping rate	95.82%
Average sequencing depth:	266.54X
Coverage >= 1X	99.36%
Coverage_at_least_4X:	98.68%
Coverage_at_least_10X:	97.89%
Coverage_at_least_20X:	97.00%

Supplementary Table 6 | Statistics of read mapping of 35 RNA-seq datasets derived from 13 adult tissues or organs of *C. farreri*.

Tissues/organs	Number of paired-end reads	Uniquely mapping ratio (%)
Striated_muscle_1 (Stmu)	10,594,953	90.20
Striated_muscle_2	14,508,215	89.58
Striated_muscle_3	11,653,026	89.59
Smooth_muscle_1 (Smmu)	12,074,884	91.75
Smooth_muscle_2	16,858,663	88.52
Smooth_muscle_3	16,912,098	90.34
Foot_1	10,999,946	80.3
Foot_2	14,210,059	79.74
Foot_3	18,615,978	83.18
Hepatopancreas_1 (Hepa)	15,955,346	76.3
Hepatopancreas_2	11,872,928	80.67
Hepatopancreas_3	7,080,147	79.49
Kidney_1 (Kidn)	17,206,931	82.67
Kidney_2	12,679,640	80.91
Kidney_3	15,551,889	80.48
Fgonad_1 (Fgon)	12,978,741	81.02
Fgonad_2	12,045,082	83.5
Fgonad_3	17,772,708	79.53
Mgonad_1 (Mgon)	14,682,133	80.13
Mgonad_2	18,323,190	79.45
Mgonad_3	11,040,841	76.63
Hemolymph_1 (Hemo)	15,143,203	82.91
Hemolymph_2	12,894,247	83.83
Hemolymph_3	16,922,568	77.7
Gill_1	19,119,031	81.65
Gill_2	19,340,526	80.76
Gill_3	16,539,933	78.62
Eye_1	16,863,537	82.96
Eye_2	11,948,076	80.52
Eye_3	17,376,242	79.38
Mantle_1 (Mant)	13,473,089	84.16
Mantle_2	12,720,957	83.08
Mantle_3	23,804,936	78.06
Cerebral ganglion (PGCG)	17,424,367	84.01
Visceral ganglion (PVG)	19,268,621	83.77

Supplementary Table 7 | BUSCO-based assessment of 10 lophotrochozoan genome assemblies based on 843 metazoan single-copy orthologs.

Species	Assembly size (Mb)	BUSCO assessment results[a]
H. robusta	239	C:75%[D:3.4%], F:10%, M:14%
C. teleta	341	C:94%[D:7.5%], F:3.6%, M:1.5%
L. anatine	433	C:91%[D:21%], F:4.7%, M:3.4%
O. bimaculoides	2,389	C:85%[D:1.8%], F:6.9%, M:7.1%
L. gigantea	366	C:93%[D:3.9%], F:4.0%, M:2.9%
C. gigas	568	C:90%[D:6.8%], F:6.4%, M:3.0%
P. fucata [Takeuchi_2016]	829	C:84%[D:4.5%], F:8.6%, M:6.5%
P. fucata [Du_2017]	991	C:79%[D:10%], F:8.4%, M:11%
C. farreri	780	C:88%[D:3.9%], F:5.5%, M:5.5%
P. yessoensis	989	C:91%[D:3.9%], F:4.2%, M:3.9%

[a] C: complete [D: duplicated], F: fragmented, M: missing.

Note, for *P. fucata*, there are two recently updated genome assemblies generated by Takeuchi et al.[53] (contig N50= 21.3 kb, total gene number= 29,353) and Du et al.[54] (contig N50= 21.5 kb, total gene number= 32,937), presenting significant improvement over the original one[55] (contig N50= 1.6 kb, total gene number= 43,760). BUSCO assessment of the gene sets from the two new assemblies suggests that Takeuchi's new gene set has slightly better quality than Du's gene set and thus was chosen in our study for further analysis.

Supplementary Table 8 | Summary statistics of the linkage map and anchored scaffolds of *C. farreri*.

Linkage group (LG)	Mapped markers	Genetic length(cM)	Marker interval(cM)	Length of anchored scaffolds (bp)
1	171	96.09	0.56	29,391,739
2	169	88.62	0.52	36,228,673
3	173	80.89	0.47	33,324,712
4	157	75.95	0.48	37,551,144
5	143	66.09	0.46	38,556,828
6	166	99.62	0.60	31,598,556
7	142	76.31	0.54	28,088,284
8	135	60.46	0.45	31,562,791
9	113	91.94	0.81	30,746,578
10	105	54.39	0.52	28,398,242
11	111	80.5	0.73	28,691,198
12	108	92.82	0.86	31,131,245
13	99	81.04	0.82	23,121,454
14	78	65.84	0.84	21,091,923
15	113	58.81	0.52	23,932,350
16	100	84.01	0.84	18,755,414
17	81	77.89	0.96	14,043,536
18	90	55.13	0.61	18,298,657
19	72	79.09	1.10	17,050,012
Total	2,236	1,465.49	0.66	521,563,336

Supplementary Table 9 | Summary of functional annotation of *C. farreri* genes.

	Number	Percent (%)
Total	28,602	100
InterPro	17,860	66.44
GO	14,014	49.00
KEGG	18,347	64.15
Swissprot	18,696	65.37
TrEMBL	24,332	85.07
Annotated	24,817	86.77
Unannotated	3,785	13.23

Supplementary Table 10 | Statistics of repeat elements in the *C. farreri* genome and four genomes of other mollusk species.

		C. farreri		*C. gigas*		*P. fucata*		*L. gigantea*		*O. bimaculoides*	
	Type	Length (bp)	% of genome	Length (bp)	% of genome	Length (bp)	% of genome	Length (bp)	% of genome	Length (bp)	% of genome
Homology-based	DNA	24,350,956	2.98	139,038,077	24.89	27,194,587	3.34	6,439,029	1.79	324,009,802	13.66
	LINE	25,194,968	3.09	17,564,585	3.14	21,979,374	2.70	8,951,872	2.49	235,726,796	9.94
	SINE	1,641,004	0.20	132,927	0.02	324,607	0.04	574,843	0.16	113,295,560	4.78
	LTR	11,575,582	1.42	15,827,973	2.83	7,394,695	0.91	5,681,209	1.58	71,401,320	3.01
	Other	4,426	0.00	551	0.00	707	0.00	652	0.00	305,870	0.01
	Unknown	333,823	0.04	2,790,831	0.50	317,808	0.04	37,087	0.01	3,519,338	0.15
De novo prediction	DNA	45,801,557	5.61	133,812,117	23.95	89,364,997	10.96	13,927,422	3.87	358,097,010	15.10
	LINE	28,374,797	3.48	9,861,054	1.77	74,939,545	9.19	6,789,942	1.89	219,081,927	9.24
	SINE	3,107,321	0.38	433,468	0.08	2572646	0.32	5299270	1.47	245,439,695	10.35
	LTR	11,371,076	1.39	5,118,548	0.92	1,706,956	0.21	979392	0.27	25,090,765	1.06
	Other	0	0.00	0	0.00	0	0.00	0	0.00	0	0.00
	Unknown	85,376,914	10.46	19,916,370	3.57	202,968,357	24.89	51,272,469	14.26	245,209,703	10.34
	Trf[a]	92,284,699	11.30	22,945,775	4.11	63,152,885	7.75	15,536,019	3.65	159,080,534	6.71
Combined total		261,830,340	32.07	193,293,205	34.60	408,113,935	50.06	95,709,712	23.04	1,160,128,281	48.92

[a] Trf stands for "Tandem repeat".

Supplementary Table 11 | Number and density of SNPs in six *C. farreri* individuals.

	Assembled[a]	RsInd1[b]	RsInd2	RsInd3	RsInd4	RsInd5
Total	4,850,636	3,523,547	3,194,680	2,653,755	2,704,112	3,222,924
Rate(%)	0.8098	0.7495	0.6871	0.7211	0.6507	0.7281
Intergenic	2,769,729	2,014,979	1,816,454	1,523,810	1,545,543	1,847,704
Rate(%)	0.8058	0.7589	0.6922	0.7335	0.6569	0.7375
Gene region	2,080,907	1,508,568	1,378,226	1,129,945	1,158,569	1,375,220
Rate(%)	0.8152	0.7374	0.6805	0.7049	0.6427	0.7160
Exon	225,313	220,691	204,340	183,551	188,671	207,264
Rate(%)	0.6804	0.6849	0.6408	0.6563	0.6334	0.6648
Intron	1,855,594	1,287,877	1,173,886	946,394	969,898	1,167,956
Rate(%)	0.8353	0.7471	0.6880	0.7152	0.6445	0.7259
Non-synonymous	NA	NA	NA	NA	NA	NA
Synonymous	NA	NA	NA	NA	NA	NA

[a]Assembled means the scallop individual used for genome sequencing and assembly.
[b]RsInd* means 5 re-sequenced individuals.

Supplementary Table 12 | Statistical significance of SNP density in the CDS regions of *Hox* genes compared to all genes in the *C. farreri* genome.

	p-value
Hox1	2.35E-03
Hox2	3.80E-02
Hox3	1.14E-02
Hox4	4.92E-02
Hox5	1.70E-07
Lox5	2.63E-04
Antp	4.90E-04
Lox4	5.09E-07
Lox2	4.11E-03
Post2	6.58E-05
Post1	1.09E-05

[a] *P* values were calculated based on the one-sided Fisher's exact test.

Supplementary Table 13 | GO enrichment analysis of gene families expanded in the *C. farreri* genome.

GO terms	Ontology	Description	GO level	Adjusted *p*
GO:0008146	F	sulfotransferase activity	5	1.12E-83
GO:0005328	F	neurotransmitter:sodium symporter activity	4	1.12E-83
GO:0006836	P	neurotransmitter transport	4	1.18E-80
GO:0044765	P	single-organism transport	3	1.61E-62
GO:0005215	F	transporter activity	2	1.22E-47
GO:0022804	F	active transmembrane transporter activity	4	4.00E-44
GO:0055085	P	transmembrane transport	4	1.23E-42
GO:0006486	P	protein glycosylation	4	7.83E-39
GO:0006810	P	transport	4	1.04E-32
GO:0016020	C	membrane	2	1.62E-31
GO:0044723	P	single-organism carbohydrate metabolic process	4	3.88E-28
GO:0016021	C	integral component of membrane	3	2.57E-27
GO:0008417	F	fucosyltransferase activity	6	3.74E-22
GO:0022857	F	transmembrane transporter activity	3	9.11E-21
GO:0004725	F	protein tyrosine phosphatase activity	8	6.45E-20
GO:0016758	F	transferase activity, transferring hexosyl groups	5	8.28E-20
GO:0008378	F	galactosyltransferase activity	6	7.37E-18
GO:0016705	F	oxidoreductase activity, acting on paired donors, with incorporation or reduction of molecular oxygen	4	1.35E-14
GO:0044699	P	single-organism process	2	3.77E-14
GO:0007155	P	cell adhesion	3	8.24E-14
GO:0030246	F	carbohydrate binding	3	1.07E-12
GO:0046906	F	tetrapyrrole binding	4	1.22E-12
GO:0006470	P	protein dephosphorylation	7	4.09E-12
GO:0016740	F	transferase activity	3	4.66E-12
GO:0020037	F	heme binding	5	1.74E-11
GO:0009055	F	electron carrier activity	2	2.67E-11
GO:0016791	F	phosphatase activity	6	3.56E-10
GO:0005506	F	iron ion binding	7	3.56E-10
GO:0015075	F	ion transmembrane transporter activity	5	2.35E-09
GO:0005507	F	copper ion binding	7	2.42E-09
GO:0008168	F	methyltransferase activity	5	7.51E-09
GO:0005975	P	carbohydrate metabolic process	4	1.57E-08
GO:0022892	F	substrate-specific transporter activity	3	4.74E-08
GO:0005230	F	extracellular ligand-gated ion channel activity	8	5.90E-08
GO:0015074	P	DNA integration	6	6.54E-08
GO:0000087	P	mitotic M phase	5	1.07E-07
GO:0009308	P	amine metabolic process	5	2.18E-07
GO:0042626	F	ATPase activity, coupled to transmembrane movement of substances	5	2.33E-07
GO:0008158	F	hedgehog receptor activity	5	2.45E-07
GO:0005044	F	scavenger receptor activity	4	5.98E-07
GO:0008131	F	primary amine oxidase activity	6	6.82E-07
GO:0016491	F	oxidoreductase activity	3	9.77E-07
GO:0001733	F	galactosylceramide sulfotransferase activity	7	1.11E-06
GO:0006388	P	tRNA splicing, via endonucleolytic cleavage and ligation	9	2.94E-06
GO:0006596	P	polyamine biosynthetic process	8	1.37E-05
GO:0016051	P	carbohydrate biosynthetic process	5	1.69E-05

GO:0008324	F	cation transmembrane transporter activity	6	2.51E-05
GO:0048038	F	quinone binding	4	3.41E-05
GO:0046914	F	transition metal ion binding	6	1.16E-04
GO:0050909	P	sensory perception of taste	8	4.37E-04
GO:0016747	F	transferase activity, transferring acyl groups other than amino-acyl groups	5	1.49E-03
GO:0008374	F	O-acyltransferase activity	6	3.25E-03
GO:0015889	P	cobalamin transport	5	3.78E-03
GO:0009593	P	detection of chemical stimulus	4	3.78E-03
GO:0055114	P	oxidation-reduction process	4	5.01E-03
GO:0016888	F	endodeoxyribonuclease activity, producing 5'-phosphomonoesters	8	5.15E-03
GO:0008898	F	S-adenosylmethionine-homocysteine S-methyltransferase activity	7	5.19E-03
GO:0016712	F	oxidoreductase activity, acting on paired donors, with incorporation or reduction of molecular oxygen, reduced flavin or flavoprotein as one donor, and incorporation of one atom of oxygen	5	5.44E-03
GO:0051260	P	protein homooligomerization	7	7.53E-03
GO:0031177	F	phosphopantetheine binding	4	1.06E-02
GO:0002755	P	MyD88-dependent toll-like receptor signaling pathway	8	2.46E-02
GO:0034130	P	toll-like receptor 1 signaling pathway	8	2.46E-02
GO:0042116	P	macrophage activation	5	2.46E-02
GO:0042495	P	detection of triacyl bacterial lipopeptide	8	2.46E-02
GO:0050707	P	regulation of cytokine secretion	5	2.46E-02
GO:0007267	P	cell-cell signaling	4	3.35E-02
GO:0005794	C	Golgi apparatus	4	4.30E-02
GO:0031419	F	cobalamin binding	5	4.37E-02

Supplementary Table 14 | Gene expression analysis of enzymes participating in glycolysis, TCA cycle, and oxidative phosphorylation pathways in the scallop *C. farreri* striated muscle (S-St), smooth muscle (S-Sm), and the adductor muscle (O-ad) in the oyster *C. gigas*.

Pathway	Gene	Enzyme	EC NO.	S-St (TPM)	S-Sm (TPM)	O-ad (TPM)	S-St vs S-Sm *p*-value[a]	S-St vs O-ad *p*-value[a]	S-Sm vs O-ad *p*-value[a]
Glycolysis	HK	Hexokinase	2.7.1.1	109.8	65.0	520.7	3.36E-05	5.73E-65	1.76E-98
	PFK	6-phosphofructokinase 1	2.7.1.11	16.9	15.3	30.1	5.46E-01	5.26E-02	9.60E-03
	FBP	Fructose-1,6-bisphosphatase I	3.1.3.11	16.1	18.6	272.3	9.27E-01	1.54E-59	1.33E-62
	ALDO	Fructose-bisphosphate aldolase	4.1.2.13	5.8	10.4	437.0	3.53E-01	2.82E-104	6.23E-111
	GAPDH	Glyceraldehyde 3-phosphate dehydrogenase	1.2.1.12	2975.5	1414.0	1108.1	1.56E-166	5.29E-195	1.08E-03
	PGK	Phosphoglycerate kinase	2.7.2.3	25.2	61.4	28.5	5.61E-04	6.52E-01	2.75E-03
	PGAM	2,3-bisphosphoglycerate-dependent phosphoglycerate mutase	5.4.2.11	524.1	286.8	29.7	1.83E-23	8.19E-114	1.41E-48
	ENO	Enolase	4.2.1.11	118.6	92.4	137.7	8.58E-03	2.33E-01	1.18E-04
	PK	Pyruvate kinase	2.7.1.40	157.4	230.9	122.8	8.36E-03	3.85E-02	1.95E-06
TCA cycle	PDH	Pyruvate dehydrogenase E1 component alpha subunit	1.2.4.1	269.5	210.3	160.6	7.94E-05	1.29E-07	1.34E-01
	DLAT	Dihydrolipoamide acetyltransferase	2.3.1.12	106.9	73.8	50.9	1.25E-03	6.59E-06	1.53E-01
	CS	Citrate synthase	2.3.3.1	30.3	28.8	428.1	5.28E-01	7.41E-89	1.17E-97
	ACO	Aconitate hydratase	4.2.1.3	106.2	92.3	384.8	7.41E-02	2.53E-38	1.12E-50
	IDH	Isocitrate dehydrogenase	1.1.1.42,1.1.1.41	991.7	681.2	217.2	3.32E-23	1.89E-118	2.38E-46
	OGDH	sucA 2-oxoglutarate dehydrogenase	1.2.4.2, 2.3.1.61	53.9	52.5	192.0	9.87E-01	4.34E-02	3.61E-02
	LSC2	Succinyl-CoA synthetase beta subunit	6.2.1.4, 6.2.1.5	224.6	231.8	124.9	3.82E-01	7.85E-08	3.13E-06
	SDH	Succinate dehydrogenase (ubiquinone) iron-sulfur subunit	1.3.5.1	366.8	351.2	637.3	3.55E-02	8.60E-18	9.35E-28
	FUM	Fumarate hydratase	4.2.1.2	21.6	16.7	58.7	2.53E-01	2.46E-05	5.75E-08
	MDH	Malate dehydrogenase	1.1.1.37	934.6	859.8	1026.3	3.04E-05	3.82E-02	2.97E-10
	ACLY	ATP citrate (pro-S)-lyase	2.3.3.8	0.7	1.0	13.1	8.75E-01	3.13E-04	2.99E-04

Oxidative phosphorylation	NDU	NADH dehydrogenase	1.6.5.3, 1.6.99.3	2493.6	2139.6	5092.9	1.17E-19	2.51E-200	0.00E+00
	DHSB	succinate dehydrogenase	1.3.5.1	366.8	351.2	637.3	3.55E-02	8.60E-18	9.35E-28
	CY	cytochrome c reductase	1.10.2.2	1427.7	1111.8	2922.8	6.60E-20	2.85E-116	2.46E-231
	COX	cytochrome c oxidase	1.9.3.1	2656.4	2233.7	2231.4	1.21E-23	1.13E-09	1.71E-04
	ATP	F-type H+-transporting ATPase	3.6.3.14	6432.5	5255	5681	2.40E-65	7.34E-12	1.11E-23

[a] *P* values were calculated by the MARS method implemented in DEGseq R package[56].

Supplementary Table 15 | Expression analysis of vertebrate-muscle marker genes in striated muscle (St) and smooth muscle (Sm) of the scallop *C. farreri* and human.

	Gene	ID	TPM		Fold change (St/Sm)	*p*-value[a]
			St	Sm		
Scallop	Myosin heavy chain, striated muscle (*Mhc*)	CF341.8	19024.5	12420.6	1.53	0
	Troponin T (*Tnnt*)	CF56167.8	17921.5	7228.5	2.48	0
	Calponin-3 (*Cnn3*)	CF55319.24	4552.2	26866.7	0.17	0
	ST-MRLC (*Mrlc*)	CF37629.19	66320.1	31724.4	2.09	0
	Troponin I (*Tnni*)	CF64639.13	10985.3	3155.7	3.48	0
	Titin (*Ttn*)	CF59337.4	38.9	43.5	0.89	0.99
	Titin (*Ttn*)	CF59337.5	104.5	297.4	0.35	1.90E-18
	Zasp	CF41309.33	577.6	1161.0	0.50	2.20E-32
	Zasp	CF41405.71	2269.4	1321.3	1.72	7.64E-83
	Zasp	CF58153.30	123.9	1620.6	0.08	7.19E-27
	Zasp	CF64013.15.1	156.5	151.9	1.03	2.10E-01
	Zasp	CF64013.17	345.9	263.5	1.31	2.14E-06
Human	Myosin heavy chain, striated muscle (*Mhc*)		4307	0.6	7178.33	0
	Troponin T (*Tnnt*)		9595.3	3.9	2460.33	0
	Calponin (*Cnn*)		1.6	3071.6	0.00	0

[a] *P* values were calculated by the MARS method implemented in DEGseq R package[56].

Supplementary Table 16 | Rhabdomeric and ciliary phototransduction related genes and their expression in the eyes of *C. farreri*.

Pathway	Gene name	geneID	eye1*	eye2	eye3
Rhabdomeric pathway	r-opsin1	CF63953.11	2.63	2.26	1.96
	r-opsin2	CF65015.35	8.51	6.15	4.02
	r-opsin3	CF65015.36	42.69	39.96	56.48
	r-opsin4	CF65015.34	1727.19	3424.46	2093.33
	Gq-protein alpha subunit	CF31469.1	101.19	169.65	93.95
	Phospholipase (PLC)	CF22817.38.1	27.35	28.34	21.15
		CF55943.26	3.46	7.88	3.09
		CF55943.25	8.53	15.98	10.33
		CF48227.3	17.27	22.25	14.24
		CF47691.35	4.04	6.64	2.91
	Protein kinase C (PKC)	CF34531.123_CF34531.126	23.9	31.01	23.9
		CF55427.11	3.17	6.65	2.08
		CF56167.43	20.9	19.84	10.54
		CF32689.7	23.7	44.33	19.87
		CF64439.88	5.29	5.95	2.32
	TRP	CF4133.1	2.41	3.16	2.96
		CF62023.33.1	31	31.9	32.13
		CF51977.10.1	2.78	3.42	2.54
		CF64439.16	0.61	1.95	0.82
Ciliary pathway	c-opsin1	CF50279.10	65.25	121.01	73.94
	c-opsin2	CF64439.21	0.12	0.45	0
	Go-opsin1	CF16535.118	0	0	0.1
	Go-opsin2	CF7665.7	1.84	0.71	1.53
	Gi(o/s)-protein alpha subunit	CF50109.22	86.57	118.52	92.2
		CF58153.38	30.2	43.83	25.3
		CF40635.6	52.8	57.76	46.35
	cGMP gated (channel alpha) nonselective	CF31569.9	0.79	0.79	0.76
		CF725573.1	0	0	0.36
		CF49817.2	0.86	1.15	0.7
		CF685457.1	0	0	0
		CF63239.14	4.9	5.77	4.04
		CF493.20	1.42	2.28	0.93
		CF15073.21.1	6.94	7.09	6.46
	cGMP gated channel alpha(potassium specific)	CF6301.20_CF6301.18	7.58	7.21	5.79
		CF35209.1	3.57	3.43	1.75
		CF16341.7	3.19	4.58	3.3
	cGMP gated channel beta	CF63239.14	4.9	5.77	4.04
	Guanyl cyclase (GC)	CF14377.10	2.54	1.05	2.08
	Adenylyl cyclase(AC)	CF60745.30	0	0	0
		CF64905.17	0.13	0.12	0

	CF724869.1	0	0.46	0
	CF61557.10	3.33	2.28	1.73
	CF13637.4	1.15	1.31	0.39
	CF11633.10_CF11633.7	24.64	44.68	30.3
PDE	CF15027.33	0.09	0	0
	CF15027.34	0	0.26	0
	CF63651.16	4.24	3.59	3.55
	CF35281.1	0	1.83	2.1
	CF35279.1	4.51	8.3	2.93
	CF57847.6	4.24	14.54	5.54
	CF64571.2	16.03	37.53	22.84
	CF12123.3.1	4.56	7.23	3.54
	CF43745.1.1	12.83	16.39	15.14
	CF28019.14	12.73	16.23	13.11

[*]Eye1-3 refer to gene expression values from eyes of three scallop individuals.

Supplementary Table 17 | Expression analysis of diff. BRPs in the juvenile and adult foot of scallops *C. farreri* (CF) and *P. yessoensis* (PY).

Gene	Color	Juvenile-TPM				Adult-foot-TPM			
		CF	PY	Fold change (CF/PY)	*p*-value	CF	PY	Fold change (CF/PY)	*p*-value[a]
Metalloproteinase inhibitor		42.4	131.0	0.3	5.72E-12	14930.7	136.2	109.6	0
Serine protease inhibitor		25.5	32.6	0.8	3.50E-01	987.2	33.8	29.2	3.33E-228
Peroxidase		0.7	0.0	NA	3.85E-01	524.8	0.0	NA	2.85E-31
Sodium/calcium exchanger		0.2	0.0	NA	6.54E-01	12293.9	0.0	NA	4.83E-239
Tenascin-X		8.8	1.3	6.7	1.29E-02	12426.8	1.4	9110.1	0
CD109 antigen		4.1	5.3	0.8	6.95E-01	1237.1	5.5	223.4	4.54E-265
Unknown protein		60.5	18.5	3.3	1.26E-06	2420.0	19.2	126.2	0
Tyrosinase		8.6	9.7	0.9	7.97E-01	2430.1	10.1	241.1	0

[a] *P* values were calculated by the MARS method implemented in DEGseq R package[56].

Supplementary Table 18 | PST concentration in eight major organs of *C. farreri*, with each organ represented by a pool of 10 individuals.

PSTs	PST concentration (ng g^{-1})							
	kidney	hepatopancreas	gonad	gill	mantle	muscle	PVCG	PVG
GTX1	1.15	7.67	2.01	6.91	10.24	0	0	0
GTX4	2.04	2.23	0.97	0.84	1.99	0.65	0	2.70
GTX2	0	1.52	0.80	2.73	2.70	0	0	21.98
GTX3	147.74	5.96	0.16	2.65	2.72	0.09	0	3.32
C1	3.06	1.42	0.19	1.14	1.60	0.42	11.55	2.61
C2	426.89	216.78	0.46	1.49	7.47	2.59	2.36	6.40
STX	0.47	1.91	0	0.39	5.82	0.60	14.49	12.64
NEO	8.65	178.78	53.69	3.52	15.96	65.71	16.90	59.89

Abbreviations: Saxitoxin (STX), N-sulfocarbamoyl toxins (C1/2), neosaxitoxin (NeoSTX) and gonyautoxins (GTX1-4).

Supplementary Table 19 | A summary of 36 transcriptome datasets of the hepatopancreas and kidney collected at 6 time points after feeding of scallops with *A. minutum*, with each data point represented by 3 individuals.

Tissue_timepoint_individual	Number of paired-end reads	Uniquely mapping ratio (%)
Kidney_0day_1	10,801,591	76.67
Kidney_0day_2	13,825,395	75.07
Kidney_0day_3	9,468,323	77.84
Kidney_1day_1	9,444,604	78.52
Kidney_1day_2	25,239,447	50.50
Kidney_1day_3	11,095,711	74.74
Kidney_3day_1	22,143,071	65.22
Kidney_3day_2	13,567,148	79.83
Kidney_3day_3	8,711,619	77.03
Kidney_5day_1	10,610,133	76.67
Kidney_5day_2	18,577,268	80.82
Kidney_5day_3	11,775,595	80.36
Kidney_10day_1	10,897,632	79.79
Kidney_10day_2	15,947,681	76.38
Kidney_10day_3	13,637,884	78.69
Kidney_15day_1	11,650,064	77.88
Kidney_15day_2	11,378,977	78.72
Kidney_15day_3	12,388,723	80.90
Hepatopancreas_0day_1	14,850,014	83.49
Hepatopancreas_0day_2	19,263,545	83.84
Hepatopancreas_0day_3	9,239,707	83.24
Hepatopancreas_1day_1	13,063,843	84.28
Hepatopancreas_1day_2	15,891,454	83.30
Hepatopancreas_1day_3	15,181,802	84.76
Hepatopancreas_3day_1	9,848,669	84.39
Hepatopancreas_3day_2	20,757,466	84.05
Hepatopancreas_3day_3	12,800,620	85.24
Hepatopancreas_5day_1	19,327,369	82.93
Hepatopancreas_5day_2	10,566,859	83.53
Hepatopancreas_5day_3	9,154,480	81.10
Hepatopancreas_10day_1	12,904,300	84.72
Hepatopancreas_10day_2	12,858,024	83.17
Hepatopancreas_10day_3	11,579,139	83.41
Hepatopancreas_15day_1	13,528,432	84.08
Hepatopancreas_15day_2	14,132,688	81.83
Hepatopancreas_15day_3	15,501,819	83.98

Supplementary References:

1. Green, M. R. & Sambrook, J. Molecular Cloning: A Laboratory Manual 4th edn, Vol.1, pp. 1.58-1.63 (Cold Spring Harbor Laboratory Press, 2012).
2. Van Nieuwerburgh, F. *et al*. Illumina mate-paired DNA sequencing-library preparation using Cre-Lox recombination. *Nucleic Acids Res*. **40**, e24 (2012).
3. Li, R. *et al.* The sequence and de novo assembly of the giant panda genome. *Nature* **463**, 311-317 (2010).
4. Huang, J. *et al*. The Jujube genome provides insights into genome evolution and the domestication of sweetness/acidity taste in fruit trees. *PLoS Genet* **12**, e1006433 (2016).
5. Harris, R.S. Improved pairwise alignment of genomic DNA, PhD Thesis. The Pennsylvania State University. (2007).
6. Li, H. & Durbin, R. Fast and accurate short read alignment with Burrows-Wheeler transform. *Bioinformatics* **25**, 1754-1760 (2009).
7. Grabherr, M.G. *et al.* Full-length transcriptome assembly from RNA-Seq data without a reference genome. *Nat Biotechnol* **29**, 644-652 (2011).
8. Simão F.A., Waterhouse R.M., Ioannidis P., Kriventseva E.V. & Zdobnov E.M. BUSCO: assessing genome assembly and annotation completeness with single-copy orthologs. *Bioinformatics* **31**, 3210-3212 (2015).
9. Wang, S., Meyer, E., McKay, J.K. & Matz, M.V. 2b-RAD: a simple and flexible method for genome-wide genotyping. *Nat Methods* **9**, 808-810 (2012).
10. Jiao, W. *et al.* High-resolution linkage and quantitative trait locus mapping aided by genome survey sequencing: building up an integrative genomic framework for a bivalve mollusc. *DNA Res* **21**, 85-101 (2014).
11. Benson, G. Tandem repeats finder: a program to analyze DNA sequences. *Nucleic Acids Res* **27**, 573-580 (1999).
12. Chen, N. Using RepeatMasker to identify repetitive elements in genomic sequences. *Curr. Protoc. Bioinformatics* **25**, 4.10.1–4.10.14 (2004).
13. Birney, E., Clamp, M. & Durbin, R. GeneWise and Genomewise. *Genome Res* **14**, 988-995 (2004).
14. Stanke, M. & Waack, S. Gene prediction with a hidden Markov model and a new intron submodel. *Bioinformatics* **19 Suppl 2**, ii215-225 (2003).
15. Majoros, W.H., Pertea, M. & Salzberg, S.L. TigrScan and GlimmerHMM: two open source ab initio eukaryotic gene-finders. *Bioinformatics* **20**, 2878-2879 (2004).
16. Korf, I. Gene finding in novel genomes. *BMC Bioinformatics* **5**, 59 (2004).
17. Trapnell, C., Pachter, L. & Salzberg, S.L. TopHat: discovering splice junctions with RNA-Seq. *Bioinformatics* **25**, 1105-1111 (2009).
18. Trapnell, C. *et al.* Transcript assembly and quantification by RNA-Seq reveals unannotated transcripts and isoform switching during cell differentiation. *Nat Biotechnol* **28**, 511-515 (2010).
19. Haas, B.J. *et al.* Automated eukaryotic gene structure annotation using EVidenceModeler and the program to assemble spliced alignments. *Genome Biol* **9**, R7 (2008).
20. Hu, X. *et al.* Cloning and characterization of tryptophan 2,3-dioxygenase gene of Zhikong scallop *Chlamys farreri* (Jones and Preston 1904). *Aquac Res* **37**, 1187-1194 (2006).
21. Dobin, A. *et al.* STAR: ultrafast universal RNA-seq aligner. *Bioinformatics* **29**, 15-21 (2013).
22. Anders, S., Pyl, P.T. & Huber, W. HTSeq--a Python framework to work with high-throughput sequencing data. *Bioinformatics* **31**, 166-169 (2015).
23. Robinson, M.D., McCarthy, D.J. & Smyth, G.K. edgeR: a Bioconductor package for differential expression analysis of digital gene expression data. *Bioinformatics* **26**, 139-140 (2010).
24. Li, H. *et al.* The Sequence Alignment/Map format and SAMtools. *Bioinformatics* **25**, 2078-2079 (2009).

25. Wang, K., Li, M. & Hakonarson, H. ANNOVAR: functional annotation of genetic variants from high-throughput sequencing data. *Nucleic Acids Res* **38**, e164 (2010).
26. Krzywinski, M. *et al.* Circos: an information aesthetic for comparative genomics. *Genome Res* **19**, 1639-1645 (2009).
27. Li, L., Stoeckert, C.J., Jr. & Roos, D.S. OrthoMCL: identification of ortholog groups for eukaryotic genomes. *Genome Res* **13**, 2178-2189 (2003).
28. Kocot KM, Citarella MR, Moroz LL & Halanych KM. PhyloTreePruner: A phylogenetic tree-based approach for selection of orthologous sequences for phylogenomics. *Evol Bioinform Online* **9**, 429-435 (2013).
29. Stamatakis A. RAxML version 8: a tool for phylogenetic analysis and post-analysis of large phylogenies. *Bioinformatics* **30**, 1312-1313 (2014).
30. Abascal F, Zardoya R & Posada D. ProtTest: selection of best-fit models of protein evolution. *Bioinformatics* **2**1, 2104-2105 (2005).
31. Yang, Z. PAML 4: phylogenetic analysis by maximum likelihood. *Mol Biol Evol* **24**, 1586-1591 (2007).
32. Posada D & Crandall KA. MODELTEST: testing the model of DNA substitution. *Bioinformatics* **14**, 817-818 (1998).
33. Hedges, S.B., Dudley, J. & Kumar, S. TimeTree: a public knowledge-base of divergence times among organisms. *Bioinformatics* **22**, 2971-2972 (2006).
34. De Bie, T., Cristianini, N., Demuth, J.P. & Hahn, M.W. CAFE: a computational tool for the study of gene family evolution. *Bioinformatics* **22**, 1269-1271 (2006).
35. Chen, S. *et al.* De novo analysis of transcriptome dynamics in the migratory locust during the development of phase traits. *PLoS One* **5**, e15633 (2010).
36. Zhang, B. & Horvath, S. A general framework for weighted gene co-expression network analysis. *Stat Appl Genet Mol Biol* **4**, Article17 (2005).
37. Shannon, P. *et al.* Cytoscape: a software environment for integrated models of biomolecular interaction networks. *Genome Res* **13**, 2498-2504 (2003).
38. Brunet, T. The evolutionary origin of bilaterian smooth and striated myocytes. *Elife* **5**, e19607 (2016).
39. Huelsenbeck, J. P. & Ronquist, F. MrBayes: Bayesian inference of phylogenetic trees. *Bioinformatics* **17**, 754-755 (2001).
40. Wang, D., Zhang, Y., Zhang, Z., Zhu, J. & Yu, J. KaKs_Calculator 2.0: a toolkit incorporating gamma-series methods and sliding window strategies. *Genomics Proteomics Bioinformatics* **8**, 77-80 (2010).
41. Wang, S. *et al.* Scallop genome provides insights into evolution of bilaterian karyotype and development. *Nat Ecol Evol* **1**, 0120 (2017).
42. Kuo, C.H., Yamagata, K., Moyzis, R.K., Bitensky, M.W. & Miki, N. Multiple opsin mRNA species in bovine retina. *Brain Res* **387**, 251-260 (1986).
43. Miao, Y. *et al.* Integration of transcriptomic and proteomic approaches provides a core set of genes for understanding of scallop attachment. *Mar Biotechnol (NY)* **17**, 523-532 (2015).
44. Lyu, Q. *et al.* Proteomic analysis of scallop hepatopancreatic extract provides insights into marine polysaccharide digestion. *Sci Rep* **6**, 34866 (2016).
45. McMillan, D.R., Kayes-Wandover, K.M., Richardson, J.A. & White, P.C. Very large G protein-coupled receptor-1, the largest known cell surface protein, is highly expressed in the developing central nervous system. *J Biol Chem* **277**, 785-792 (2002).
46. Bricelj, V.M. *et al.* Sodium channel mutation leading to saxitoxin resistance in clams increases risk of PSP. *Nature* **434**, 763-767 (2005).
47. Zakon, H.H. Adaptive evolution of voltage-gated sodium channels: the first 800 million years. *Proc Natl Acad Sci U S A* **109 Suppl 1**, 10619-10625 (2012).
48. Jost, M.C. *et al.* Toxin-resistant sodium channels: parallel adaptive evolution across a complete gene family. *Mol Biol Evol* **25**, 1016-1024 (2008).
49. Choudhary, G., Yotsu-Yamashita, M., Shang, L., Yasumoto, T. & Dudley, S.C., Jr. Interactions of the C-11 hydroxyl of tetrodotoxin with the sodium channel outer

vestibule. *Biophys J* **84**, 287-294 (2003).
50. Kontis, K.J. & Goldin, A.L. Site-directed mutagenesis of the putative pore region of the rat IIA sodium channel. *Mol Pharmacol* **43**, 635-644 (1993).
51. Wu, Z. *et al*. The research of HPLC-MS/MS determination method of paralytic shellfish toxins. *T Oceanol Limn* **4**, 66-72 (2013).
52. Zhao, C., Zhang, T., Zhang, X., Hu, S. & Xiang, J. Sequencing and analysis of four BAC clones containing innate immune genes from the Zhikong scallop (*Chlamys farreri*). *Gene* **502**, 9-15 (2012).
53. Takeuchi, T., *et al*. Bivalve-specific gene expansion in the pearl oyster genome: implications of adaptation to a sessile lifestyle. *Zool Lett* **2**, 3 (2016).
54. Du, X., *et al*. The pearl oyster *Pinctada fucata martensii* genome and multi-omic analyses provide insights into biomineralization. *GigaScience* **6**, 1-12 (2017).
55. Takeuchi, T., *et al*. Draft genome of the pearl oyster *Pinctada fucata*: a platform for understanding bivalve biology. *DNA Res* **19**,117-130 (2012).
56. Wang, L., Feng, Z., Wang, X., Wang, X. & Zhang, X. DEGseq: an R package for identifying differentially expressed genes from RNA-seq data. *Bioinformatics* **26**, 136-138 (2010).
