# Supplementary material for: Scallop genome reveals molecular adaptations to semi-sessile life and neurotoxins
Source: Nat Commun. 2017 Nov 23;8:1721. doi: 10.1038/s41467-017-01927-0 (PMC5700196; doi:10.1038/s41467-017-01927-0)
Supplement: Supplementary file 2 — Description of Additional Supplementary Files [file 41467_2017_1927_MOESM2_ESM.pdf]

**Description of Additional Supplementary Files**

File Name: Supplementary Movie 1
Description: Swimming activity of Zhikong scallop (*Clamys farreri*). The scallop fully extends upper and lower velar lobes first, and then swims by fast clapping the shell valves to create jets. Lift is generated by the angle of attack of the valves through the water.

File Name: Supplementary Data 1
Description: 270 Gene families significantly expanded in the *C. farreri* genome compared to other bivalve (*C. gigas* and *P. fucata*) genomes.

File Name: Supplementary Data 2
Description: Module enrichment analysis of muscle overrepresented genes.

File Name: Supplementary Data 3
Description: Summary of transcription factor coexpression network in the *C. farreri* adductor muscle-related module (M3).

File Name: Supplementary Data 4
Description: Summary and characterization of scallop byssal proteins identified by mass spectrometric analysis of adhesive byssal plaques of *C. farreri*.

File Name: Supplementary Data 5
Description: Temporal profiles of PSTs in six organs of *C. farreri* during exposure to toxic *A. minutum*.

File Name: Supplementary Data 6
Description: Statistical significance of PST profile changes (compared to 0-day) in six organs of *C. farreri* during exposure to toxic *A. minutum*.

File Name: Supplementary Data 7
Description: Identification of toxin-response modules in the *C. farreri* kidney and hepapopancreas during exposure to toxic *A. minutum*.

File Name: Supplementary Data 8
Description: Go annotations of toxin-response modules in the *C. farreri* kidney.

File Name: Supplementary Data 9
Description: Go annotations of toxin-response modules in the *C. farreri* hepatopancreas.

File Name: Supplementary Data 10
Description: Distribution and toxin-response significance of cytosolic sulfotransferase (*SULT*) genes in the gene network of the *C. farreri* kidney.
